# Single cell analysis revealed that two distinct, unique CD4^+^ T cell subsets were increased in the small intestinal intraepithelial lymphocytes of aged mice

**DOI:** 10.3389/fimmu.2024.1340048

**Published:** 2024-01-22

**Authors:** Yuki Yonemoto, Yasuhiro Nemoto, Ryo Morikawa, Nana Shibayama, Shigeru Oshima, Takashi Nagaishi, Tomohiro Mizutani, Go Ito, Satoru Fujii, Ryuichi Okamoto

**Affiliations:** ^1^Department of Gastroenterology and Hepatology, Graduate School, Tokyo Medical and Dental University (TMDU), Tokyo, Japan; ^2^Institute of Research, Tokyo Medical and Dental University (TMDU), Tokyo, Japan; ^3^Department of Advanced Therapeutics for Gastrointestinal Diseases, Tokyo Medical and Dental University (TMDU), Tokyo, Japan; ^4^Advanced Research Institute, Tokyo Medical and Dental University (TMDU), Tokyo, Japan

**Keywords:** intraepithelial lymphocyte, T cell aging, intestinal immunity, CD4 T cell, scRNAseq

## Abstract

Recent advances in research suggest that aging has a controllable chronic inflammatory disease aspect. Aging systemic T cells, which secrete pro-inflammatory factors, affect surrounding somatic cells, and accelerate the aging process through chronic inflammation, have attracted attention as potential therapeutic targets in aging. On the other hand, there are few reports on the aging of the intestinal immune system, which differs from the systemic immune system in many ways. In the current study, we investigated the age-related changes in the intestinal immune system, particularly in T cells. The most significant changes were observed in the CD4^+^ T cells in the small intestinal IEL, with a marked increase in this fraction in old mice and reduced expression of CD27 and CD28, which are characteristic of aging systemic T cells. The proliferative capacity of aging IEL CD4^+^ T cells was significantly more reduced than that of aging systemic T cells. Transcriptome analysis showed that the expression of inflammatory cytokines was not upregulated, whereas *Cd8α*, NK receptors, and Granzymes were upregulated in aging IEL CD4^+^ T cells. Functional analysis showed that aging IEL T cells had a higher cytotoxic function against intestinal tumor organoids *in vitro* than young IEL T cells. scRNAseq revealed that splenic T cells show a transition from naïve to memory T cells, whereas intestinal T cells show the emergence of a CD8αα^+^CD4^+^ T cell fraction in aged mice, which is rarely seen in young cells. Further analysis of the aging IEL CD4^+^ T cells showed that two unique subsets are increased that are distinct from the systemic CD4^+^ T cells. Subset 1 has a pro-inflammatory component, with expression of *IFNγ* and upregulation of NFkB signaling pathways. Subset 2 does not express *IFNγ*, but upregulates inhibitory molecules and nIEL markers. Expression of *granzymes* and *Cd8a* was common to both. These fractions were in opposite positions in the clustering by UMAP and had different TCR repertoires. They may be involved in the suppression of intestinal aging and longevity through anti-tumor immunity, elimination of senescent cells and stressed cells in the aging environment. This finding could be a breakthrough in aging research.

## 1 Introduction

Today, the world is facing an unprecedented super-aging society, and medical and social countermeasures are an urgent issue (1). Several mechanisms have been proposed to explain aging, including limits on programmed cell division due to telomeres and other mechanisms (2), impaired gene repair mechanisms due to the accumulation of errors at the cellular level (3), mitochondrial degradation due to reactive oxygen species or oxidative stress (4–8), and stem cell deterioration (9), although these are not yet fully understood (10).

In addition to these mechanisms, the immune system plays an important role in the aging process by promoting it through chronic inflammation (11, 12). In immune aging, which begins in the 20s (13), the aging of long-lived T cells is more important than that of short-lived myeloid cells such as granulocytes, monocytes, and macrophages. At the individual level of aging, thymic atrophy disrupts the supply of fresh naive T cells, and antigen exposure leads to an increase in effector/memory cells (14). At the cellular level, it is also known that senescent T cells, like other somatic cells, have a low proliferative capacity and secrete pro-inflammatory humoral factors, such as TNF-α/IFN-γ (this phenotype is termed senescence-associated secretory phenotype; SASP) (14–18). In mice in which senescence was specifically induced in T cells, age-related changes such as atherosclerosis were accelerated (4), and administration of a peptide vaccine targeting senescent T cells ameliorated the pathology of a mouse model of diabetes (18), suggesting that senescent T cells are an important therapeutic target in systemic aging.

The gut is the one of the largest reservoirs of immune cells, particularly T cells. The intestinal lumen contains a large number of foreign antigens such as dietary antigens and gut microbiota. The intestinal lumen fulfils the conflicting roles of tolerating harmless antigens and inducing inflammation in response to harmful pathogens from the sea of antigens (19, 20). The intestinal tract therefore has a highly developed and unique intestinal mucosal immune system that is distinct from the systemic immune system. The intestinal immune system has its own lymphoid tissues such as gut-associated lymphoid tissue; GALT, and in addition to the conventional CD4 and CD8^+^ T cells, there are many other unique fractions such as γδT cells, CD8αβ^-^CD4^-^TCRαβ^+^ T cells, and CD8αα^+^ T cells that are rarely found in peripheral blood. The intestinal immune system is the first line of defense against foreign antigens and maintains the homeostasis of the organism by maintaining a delicate balance between inflammation and tolerance (19, 20).

The intestinal tract is a relatively unaffected organ by aging. For example, progeria syndrome causes phenotypes in the skin, hair, cardiovascular system, brain, etc., but the gut is less affected (21). On the other hand, colorectal cancer and gastrointestinal infections increase with age, while inflammatory bowel diseases; IBD and allergies decrease, suggesting that some changes are occur in the intestinal immune system (22), but there are few reports on the phenotype of intestinal aging. Furthermore, while immune aging has been extensively studied in the systemic immune system, there are few reports on age-related changes in the intestinal immune system.

In this study, we investigated the role of the intestinal immune system in aging by analyzing the qualitative, quantitative, and functional changes associated with aging in the intestinal immune system, in particular in T cells, which are long-lived and important therapeutic targets in the aging process, and by comparing them with aging T cells in the systemic immune system.

## 2 Materials and methods

### 2.1 Mice

We selected 5- to 9-weeks old and 18-months old male mice as the young and aged models based on previous papers (23–26). The general condition and organ appearance of aged mice were observed before the experiments, and mice with obvious diseases were excluded and not used in the studies. C57BL/6 mice were purchased from Jackson Laboratory or Charles River or CLEA in Japan. Both male and female mice were used for experiments. All mice were maintained under specific pathogen-free conditions in the Center for Experimental Animals in Tokyo Medical and Dental University (TMDU). All experiments were approved by animal study committees and performed in accordance with institutional guidelines and Home Office regulations.

### 2.2 Antibodies

The following antibodies were used: anti-mouse CD3e-FITC, -PerCP-Cy5.5, or -APC-Cy7 (clone 145-2C11; BioLegend), CD4-APC or -PE-Cy7 (clone RM4-5; BioLegend), anti-mouse CD8α-Alexa Fluor 488 (clone 53-6.7; BioLegend), anti-mouse CD8β-PE (clone YTS156.7.7; BioLegend), anti-mouse CD27-Brilliant Violet 510 (clone LG.3A10; BioLegend), anti-mouse CD28-Brilliant Violet 421 (clone 37.51; BioLegend), anti-mouse CD45- Brilliant Violet 421 (clone 30-F11; BioLegend), anti-mouse CD274 (PD-L1)-PE (clone 10F.9G2; BioLegend), anti-mouse epithelial cell adhesion molecule (EpCAM)-APC (clone G8.8; BioLegend), anti-mouse MHC-II-FITC (clone M5/114.15.2; eBioscience), anti-mouse TCRβ- APC-Cy7 (clone H57-597; BioLegend) or anti-mouse γδTCR -PE-Cy7 (clone GL3; BioLegend). For scRNAseq analysis, anti-mouse Hashtags 1 to 4 (M1/42; 39-F11, Biolegend, 155861, 155863, 155865, 155867) were used.

### 2.3 Isolation of mononuclear cells from murine organs

To isolate SP, MLN, and PP cells, each organ was mashed and passed through a nylon mesh. To lyse red blood cells among splenocytes, Ack buffer was used. To isolate small intestinal IELs or LPLs, we used modified protocols in previous reports (27). After the removal of PPs, a half-length of the distal ileum, proximal ileum, distal jejunum, and proximal jejunum was opened longitudinally, washed with Hank’s balanced salt solution (HBSS), and cut into small pieces. Dissected mucosae were gently inverted (50–60 rpm) on a rotator for 10–15 min at 37°C in 40 ml HBSS with 2 mM EDTA. Then, the supernatant and remaining mucosae were separated with a nylon mesh. The supernatant was centrifuged and resuspended with a 40% isotonic Percoll (GE Healthcare) solution and then subjected to Ficoll-Hypaque density gradient centrifugation (40%/75%). Collected cells were IELs. Remaining mucosae were gently inverted (50–60 rpm) on the rotator for 15–20 min at 37°C in 40 ml HBSS with 0.5 mg/ml collagenase D (Roche) and 25 μg/ml DNase I (Roche). Then, they were filtered, centrifuged, and separated with the Percoll system as described above. To isolate colonic mucosal cells, the same method was used, but the incubation time in EDTA and for digestion was 60 min.

### 2.4 Flow cytometric analysis and cell sorting

In some experiments, CD4^+^ cells or CD45^+^ cells were enriched with CD4 (L3T4) microbeads or CD45 microbeads (Miltenyi Biotec) according to official protocols before the cell sorting by flow cytometer. To stain surface molecules, the single cell suspension isolated from each organ was incubated with specific antibodies for 20 min at 4°C. For analysis, cells were resuspended with PBS and analysed by a FACS Canto II (BD Bioscience). Data were analysed by FlowJo software (FlOWJO LLC). For cell sorting, stained cells were sorted using a FACS Melody (BD Bioscience). More than 98% purity of each fraction was confirmed by post-sort purity checking with the FACS Canto II.

### 2.5 Microarray analysis

SI-IELs and SP cells were collected from 5-weeks old or 87weeks old C57BL/6 mice, stained with anti- CD3e-PerCP-Cy5.5 and anti-CD4-PE-Cy7, and then sorted by the FACS Melody as the CD4(CD3^+^CD4^+^) fractions. More than 98% purity of each fraction was confirmed by analysing post-sorted cells with the FACS Canto II. Total RNA was collected from each fraction with a RNeasy Mini Kit (QIAGEN). Microarray analysis was outsourced to KAMAKURA TECHNO-SCIENCE INC. Samples were analysed with a 3D gene chip (Mouse oligo 24k). Enrichment analysis was performed with DAVID (https://david.ncifcrf.gov/). Enriched genes were defined by more than 2-fold change and more than 100 signals after the global normalization.

### 2.6 Cell proliferation assay

1 x 10^5^ of sort-purified IEL- or SP CD4^+^ cells were labelled with 2.5μM CFSE (CellTrace CFSE Cell Proliferation Kit, ThermoFisher SCIENTIFIC), and then stimulated with PMA+ionomycin PMA+ionomycin (PMA 50ng/ml, ionomycin 500ng/ml) in the complete medium with 100 U/mL recombinant human IL2 (Roche), 50 ng/mL mouse IL7 (Peprotech), and 50 ng/mL mouse IL15 (Peprotech) under the condition of 37°C, 5% CO2. After 72 hours, cells were harvested and stained with appropriate antibodies and analyzed by FACS Canto II (BD Bioscience).

### 2.7 RT-qPCR

Total RNA from SI-IELs and SP from 5 to 6-weeks old or 80 to 95 weeks-old C57BL/6 mice was isolated as described above and converted to cDNA using QuantiTect reverse transcription kit (QIAGEN) according to the manufacturer’s instructions. Real-time PCR was performed with QuantiTect SYBRgreen PCR kits (QIAGEN) using the StepOnePlus real-time PCR system and StepOne software (Thermo Fisher Scientific). Gene expression was normalized to the housekeeping gene β-actin and was expressed as the relative expression. Primers used for RT-qPCR are listed in **Supplementary Table 1**.

### 2.8 Preparation of small intestinal organoids

Crypts were isolated from the small intestine of male WT mice aged 7–11 weeks or small intestinal tumors of female APC^min^ mice aged 21 weeks as previously described (27). One mouse was used for each isolation. The crypts were embedded in Matrigel (Corning). Advanced DMEM/F12 (Gibco) containing 50 ng/mL mEGF (Peprotech), 100 ng/mL mNoggin (R&D Systems), 100 U/mL penicillin/streptomycin (Nacalai), 10 mM HEPES (Nacalai), 2 mM GlutaMAX-1 (Gibco), 1 mM N-acetylcysteine (Sigma), 1×N2 supplement (Gibco), 1×B27 supplement (Gibco) and 10 μM Y-27632 (Wako) were added to each well (complete medium). Organoids from APC^min^ mice were passaged at least twice before co-culture with IECs.

### 2.9 Co-culture of IELs and small intestinal tumor organoids

We followed a modified protocol from a previous report (27). Briefly, intestinal organoids were cultured for 2 days prior to the co-culture with IELs. On the first day of co-culture, SI-IELs collected from the WT mice aged 9-weeks old or 89 weeks old were stained with anti-mouse CD3e-APC-Cy7, anti-mouse CD4-PE-Cy7, and the CD3^+^ (CD3^+^) and CD4^+^ (CD3^+^ CD4^+^) -IELs were sorted using FACSMelody (BD Bioscience). Cultured organoids released from Matrigel were washed and counted. We combined 200 organoids and 2.0 × 10^5^ IELs and centrifuged the samples for 3 min at 200 g. In the control group, the same number of organoids as the co-culture group was centrifuged. The pellet was suspended in 20 μL of Matrigel and placed in 24-well plates. After Matrigel polymerization, 500 μL of the complete medium with 100 U/mL recombinant human IL-2 (Roche), 10 ng/mL mouse IL-7 (Peprotech) and 10 ng/mL mouse IL-15 (Peprotech) were supplemented. The medium was refreshed every 1–2 days. Images of organoids were taken with a BZ-X710 microscope (Keyence).

### 2.10 Cell count of IELs and organoids

Organoids and IELs were collected and centrifuged for 3 min at 100 g in experiments. The pellet was shaken (500 rpm) in the trypLE express (Invitrogen) for 15 min at 37°C. After pipetting, cells were centrifuged for 3 min at 400 g, and the pellet was suspended with RPMI-medium (Sigma). Numbers of live single epithelial cells and IELs were counted with FACSCanto II (BD Bioscience) and Count Bright Absolute counting Beads (Invitrogen).

### 2.11 Single-cell 5’ transcription and T cell receptor sequencing

SI-IELs or SP cells were collected and pooled from young (6-weeks old) or old C57BL/6 mice (80-weeks old), the cells from 3 young mice and 3 old mice were mixed and pooled as young SP, young IEL, old SP and old IEL. CD45^+^ cells were enriched from each group using CD45 microbeads (Miltenyi Biotec), followed by cell hashing with TotalSeq-C anti-mouse hashtag antibodies (hashtag1-young IEL, hashtag2-old IEL, hashtag3-young SP, hashtag4-old SP). After the Fc blocking, cells were stained with anti-CD3e-FITC mAbs and anti-CD4-APC mAbs, and the CD3^+^ cells and CD3^+^CD4^+^ cells were sorted from each group by FACSMelody. Dead cells removal was performed with 7-AAD. To enrich CD4^+^ cells, which are rare population in young IEL, CD3^+^ cells and CD3^+^CD4^+^ cells were mixed as 1:1 in young and old IEL. The single cell suspension was stained with Trypan Blue and the number of cells was determined by manual cell count.

The following procedures were outsourced to AZENTA, GENEWIZ (Tokyo, Japan). Cell suspension (6,000 cells each) was loaded in a 10x Chromium microfluidics system based on the manufacturer’s guidelines. One set of libraries were obtained from the 10x loaded samples: a 5’ gene expression messenger RNA library and a single-cell TCR library, using primers for amplification as per the manufacturer’s instructions. Library were pooled together and run separate lanes of 150 base-paired, paired-end, flow cell using the Illumina Novaseq6000. 5668 cells for young SP, 5538 cells for old SP, 4348 cells for young IEL and 5811 cells for old IEL were sequenced and reported in the datasets.

The Cell Ranger software (10x Genomics) was used to perform barcode counting and unique molecular identifier counting after filtering and alignment to the mouse/mm10 reference genome to generate the feature-barcode matrix and determine clusters. Dimensionality reduction was performed using principal component analysis, and first ten principal components were used to generate clusters by K-means algorithm and graph-based algorithm, respectively. Data analysis was performed through the Loupe Cell Browser software (10x genomics).

Next, the differential expression genes for each cluster were imported into Metascape (https://metascape.org/) for Kyoto Encyclopedia of Gene and Genomes (KEGG) pathway analysis was performed with a false discovery rate (FDR) <0.05 as the cut-off value.

We used the Loupe V(D)J browser to analyse the TCR clonotypes, and the V and J genes.

### 2.12 Statistical analysis

The exact number of samples is described in the figure legends. Statistical analyses were performed using GraphPad Prism 10 for Windows 64-bit (GraphPad Software). Normality of the distribution of results was examined in each group. Differences between two groups were assessed using Student’s t-test for equal variance and Welch’s t-test for unequal variance. Non-parametric tests, such as the Mann-Whitney U test, were used for comparison between two groups without normality. For comparison among 3 or more groups was evaluated with one-way ANOVA followed by parametric multiple comparisons test. For comparison among 3 or more groups without normality, one-way ANOVA followed by non-parametric test was used. Results are expressed as the mean ± S.E.M. Differences were considered significant at P < 0.05.

## 3 Results

### 3.1 CD27^-^CD28^-^CD4^+^ T cells increased in the small intestine of aged mice

**Figure 1:**
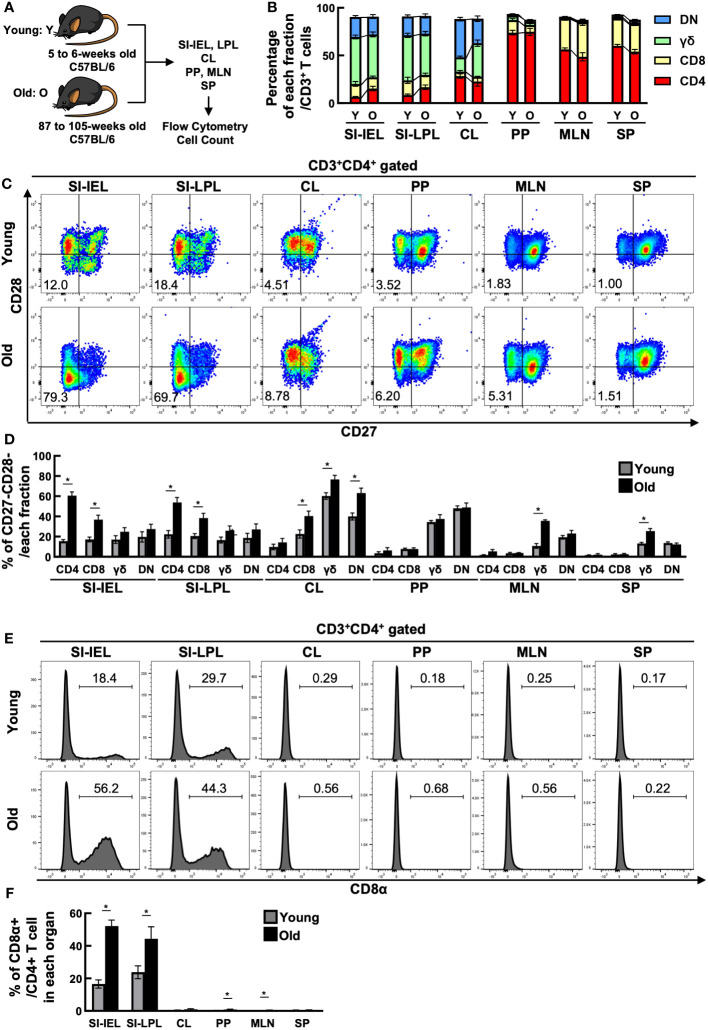
CD4^+^ CD27^-^ CD28^-^ CD8α^+^ T cells increased in the small intestine of aged mice. **(A)** Experimental design. T cells in the spleen (SP), mesenteric lymph nodes (MLN), Peyer’s patches (PP), small intestinal IELs (IEL), small intestinal LPLs (LPL), and colon (CL) of young mice (5-6 weeks old) and old mice (87-105 weeks old) were analyzed. Pooled data from 5 similar independent experiments were analyzed (n=7-11 mice/group). In this figure, “Y” indicates young mice and “O” indicates old mice. **(B)** Changes in the percentage of each fraction of T cells in each organ between young and old mice. Graph shows mean ± SEM. **(C)** Expression of CD27 and CD28 on CD4^+^ T cells in each organ of young and old mice. Representative dot plots are shown. The numbers in each plot reflect the percentage of CD27^-^CD28^-^ cells. **(D)** Percentage of CD27^-^CD28^-^ cells in each T-cell fraction of each organ from young and old mice. Graph shows mean ± SEM. *P<0.05. **(E)** Expression of CD8α on each T-cell fraction in each organ of young and old mice. Representative histograms are shown. The number on each histogram reflects the percentage of CD8α^+^ cells. **(F)** Percentage of CD8α^+^ cells in each fraction of each organ from young and old mice. Graphs show mean ± SEM. ∗P < 0.05.

To investigate the quantitative changes in T cells with age, we examined changes in T cell fractionation in the spleen (SP), mesenteric lymph nodes (MLN), Peyer’s patches (PP), small intestinal IELs (IEL), small intestinal LPLs (LPL), and colon (CL) of young mice (5-6 weeks old) and old mice (87-105 weeks old) (**Figure 1A**). T cells were CD3^+^TCR^+^ cells, which included the following fractions: CD4^+^ T cells (CD3^+^CD4^+^CD8β^-^TCRβ^+^TCRγδ^-^), CD8^+^ T cells (CD3^+^CD4^-^CD8β^+^TCRβ^+^TCRγδ^-^), DNT; double negative T cells (CD3^+^CD4^-^CD8β^-^TCRβ^+^TCRγδ^-^), and γδ T cells (CD3^+^TCRβ^-^TCRγδ^+^). As seen in **Figure 1B**, SP, a reservoir organ for systemic T cells, showed little fractional change, whereas MLN showed a decrease in CD4^+^ T cells and an increase in CD8^+^ T cells. Interestingly, the greatest change was seen in the IEL. The major fraction, γδ, DNT and CD8^+^ T cells showed little change, whereas the minor fraction, CD4^+^ T cells, more than doubled (6.55 ± 0.570 to 15.4 ± 2.35). Similar changes were observed in LPL.

As shown in **Figures 1C, D**, IEL CD4^+^ T cells showed a marked downregulation of CD27 and CD28, co-stimulatory molecules known to be downregulated with age, and a marked increase in the proportion of CD27^-^CD28^-^ cells (15.5 ± 1.60→60.5 ± 3.74). LPL CD4^+^ T cells also showed a similar increase in CD27^-^CD28^-^ cells. CD27^-^CD28^-^ cells were increased in both IEL and LPL CD8^+^ T cells, but the greatest change was seen in CD4^+^ T cells, with a 37-fold increase in cell number (**Supplementary Figure 1**). On the other hand, CD27^-^CD28^-^ cells were only partially altered in SP, MLN and PP.

The intestinal tract is known to contain many unique T cells expressing CD8αα homodimers rarely found in the systemic immune system, but interestingly, the percentage of CD8αα^+^ cells was also increased in the small intestinal IEL, LPL CD4^+^ T cell fraction of aged mice (**Figures 1E, F**).

### 3.2 Old IEL-CD4^+^ T cells had a much lower cell proliferative capacity than young IEL-CD4^+^ or old SP-CD4^+^ T cells

As described above, the number of CD4^+^ T cells in the small intestinal mucosa doubled with age, and surface markers suggested that CD27^-^CD28^-^ had characteristics as aging T cells. On the other hand, the expression rate of CD8αα, a marker specific for intestinal T cells, also increased, suggesting that they may have different characteristics from those of the systemic immune system.

We therefore focused on IEL CD4^+^ T cells to analyze functional changes associated with aging. Since senescent T cells in systemic immunity are known to express senescence markers, have low proliferative capacity, and secrete inflammatory humoral factors such as TNF-α, IFN-γ, and other cytokines, a phenotype known as SASP, we investigated these points.

**Figure 2:**
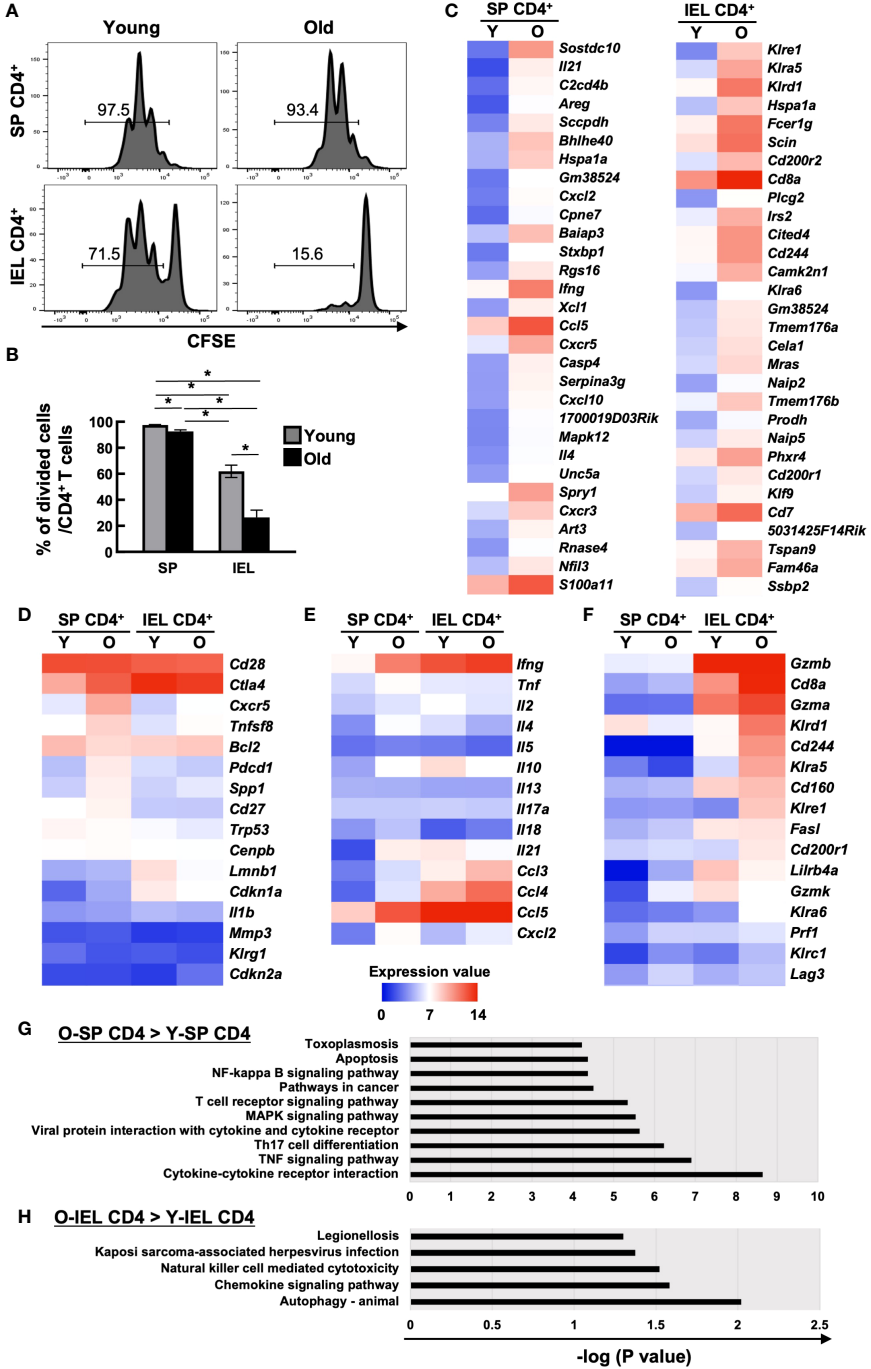
Comparison of cell proliferation capacity and comprehensive transcriptional analysis of systemic and intestinal T cells between young and old mice. **(A, B)** The proliferation capacity of SP CD4^+^ T cells and IEL CD4^+^ T cells from young (8-weeks old) and old mice (85-86-weeks old) was assessed by CFSE assay. Sort-purified IEL- or SP CD4^+^ cells were labelled with CFSE, and stimulated with PMA+ionomycin in the complete medium with IL-2, IL-7, and IL-15 for 72 hours. Pooled data from 2 similar independent experiments were analyzed (n=6/group). **(A)** Expression of CFSE after the 72 hours of stimulation. Representative histograms are shown. The number on each histogram is the percentage of CFSE-negative divided cells. **(B)** Percentage of CFSE-negative divided cells in CD4^+^ T cells of SP or IELs from young (8-weeks old) and old (85- to 86-weeks old) mice. Graph shows mean ± SEM. *P<0.05. **(C-H)** Microarray analysis of sort purified CD4 IELs or SP T cells in young (5-weeks old) or old (89-weeks old) mice. The expression value shows the log2 of the signals after global normalization. **(C)** Heat maps of the top 30 genes enriched in old SP CD4^+^ T cells (left column) and old IEL CD4^+^ T cells (right column) compared to young ones. **(D)** Heat maps of senescent associated genes in young and old SP or IEL CD4^+^ T cells. **(E)** Heat map of cytokine or chemokine genes in young and old SP or IEL CD4^+^ T cells. **(F)** Heat maps of genes of NK or cytotoxic markers or IEL markers in young and old SP or IEL CD4^+^ T cells. **(G)** KEGG pathway analysis of genes enriched in old SP CD4^+^ T cells compared to young SP CD4^+^ T cells. **(H)** KEGG pathway analysis of genes enriched in old IEL CD4^+^ T cells compared to young IEL CD4^+^ T cells.

SP CD4^+^ and IEL CD4^+^ T cells from young and aged mice were sorted by flow cytometry, labelled with CFSE and stimulated with PMA+ionomycin, and cell division after 72 hours was assessed by flow cytometry. Although IEL have been reported to have a lower proliferative capacity than systemic T cells, even the positive control IEL CD4^+^ T cells from young mice showed little cell division under normal conditions (data not shown). When IL-2, IL-7, and IL-15 were added as T cell maintenance factors, as shown in **Figures 2A, B**, SP CD4^+^ T cells showed only a slight decrease in cell division with age (97.5 ± 0.29→92.7 ± 1.05), whereas IEL-CD4 showed a marked decrease in proliferative capacity (62.0 ± 4.82→26.5 ± 5.60).

### 3.3 Comprehensive transcriptional analysis of SP CD4^+^ and IEL CD4^+^ T cells revealed a unique change in the characteristics of IEL CD4^+^ T cells with age

As mentioned above, IEL CD4^+^ T cells from aged mice differed from systemic CD4^+^ T cells in many ways, suggesting that they have completely different characteristics from the previously reported aging T cells. Therefore, we performed a comprehensive transcriptional assay as a further investigation. SP CD4^+^ and IEL CD4^+^ T cells from young and aged mice were sorted by flow cytometer and total RNA was collected for microarray analysis of expressed genes.

There was no marked upregulation of known senescence markers in old IEL CD4^+^ T cells (**Figures 2C, D**). As previously reported, SP CD4^+^ T cells from aged mice showed increased expression of inflammatory cytokines such as *Tnf, Ifng, Il18, Il21*, and *Il4*, whereas IEL-CD4^+^ cells showed similar levels of *Infg* and *Tnf* expression as young mice, but low levels of *Il21*, and *Il4* expression. Interestingly, the expression of *Il10*, an inhibitory cytokine, was high in IEL CD4^+^ from young mice, but decreased with age. On the other hand, the expression of chemokines such as *Ccl3, Ccl4*, and *Cxcl2* was increased as in the spleen (**Figures 2C, E**). Interestingly, the expression of *Cd8a* and NK receptors such as*, Klrd1, and Cd244*, which are rarely expressed on SP CD4^+^ cells, was upregulated, and these cells had features similar to gut-specific fractions such as γδ T cells and DNT cells. They also had unique features that differed from those of systemic senescent T cells, such as high expression of cytotoxic markers such as *Gzma* (**Figures 2C, F**).

KEGG pathway analysis revealed that although the NK-kB signaling, TCR signaling, TNF signaling, Th17, and cytokine-cytokine receptor pathways were enriched in old SP CD4^+^ T cells compared to young SP CD4^+^ T cells, the NK cell-mediated cytotoxicity and chemokine signaling pathways were enriched in old IEL CD4^+^ T cells compared to young IEL CD4^+^ T cells (**Figures 2G, H**).

To validate the microarray results, qPCR of some representative genes were performed. As shown in **Supplementary Figure 2**, we could confirm that higher expression of *Cd8a*, *Gzma*, *Cd200r2*, *Fcer1g and Lag3* in old IEL-CD4 and no age-related increase of *Ifng and Ctla4* in IEL-CD4.

### 3.4 Analysis of anti-tumor cytotoxic function of bulk-old IEL CD4^+^ T cells

**Figure 3:**
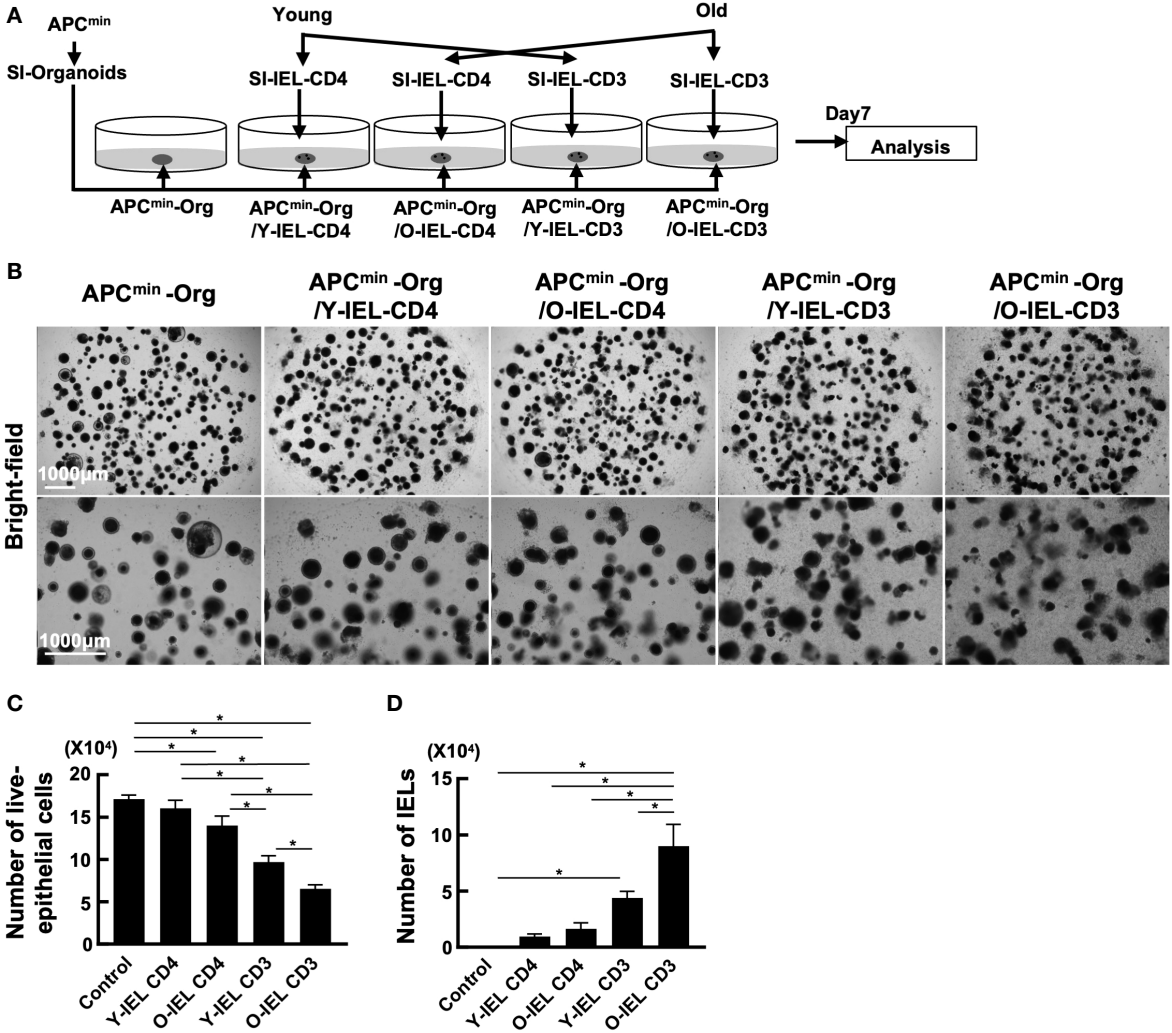
Analysis of anti-tumor cytotoxic function of bulk-old IEL CD4^+^ T cells. To investigate the role of old IEL CD4^+^ T cells in intestinal tumor immunity, we examined their anti-tumor cellular immunity *in vitro*. Pooled data from 2 similar independent experiments were analyzed (n=6 mice/group). “Org” means organoid in this figure. **(A)** Experimental design of this experiment. Organoids were generated from intestinal tumors of APC^min^ mice, and co-cultured with CD4^+^ T cells and CD3^+^ T cells from SP and IEL of young and old mice. After 7 days of co-culture, they were harvested, and the numbers tumor cells and effector T cells were assessed by flow cytometry. **(B)** Representative images of organoids and IELs after 7 days of co-culture. **(C)** The number of live epithelial cells (7-AAD^-^EpCAM^+^CD45^-^ cells) collected from organoids in each group. Graphs show mean ± SEM. ∗P < 0.05. **(D)** Number of IELs (7-AAD^-^EpCAM^-^CD45^+^ cells) in each group (n = 6). Graphs show mean ± SEM. ∗P < 0.05.

As mentioned above, the incidence of food allergy and IBD is known to decrease with age, while the incidence of colorectal tumors increases. Transcriptional analysis showed increased expression of cytotoxic genes such as NK markers and Granzyme A in intestinal IEL CD4^+^ T cells in aged mice, suggesting that intestinal CD4^+^ T cells in aging individuals may respond to the increased tumor risk by increasing their cytotoxic activity. To investigate the role of old IEL CD4^+^ T cells in intestinal tumor immunity, we examined their anti-tumor cellular immunity *in vitro*. In our previous report, we successfully recapitulated the anti-tumor cellular immune response of IELs *in vitro* by co-culturing IELs with intestinal tumor-derived organoids (27). In the present study, we used a similar model to examine the anti-tumor immune potential of old IEL CD4^+^ T cells. Organoids were generated from intestinal tumors of APC^min^ mice, a spontaneous model of multiple intestinal tumors, and co-cultured with CD4^+^ T cells and CD3^+^ T cells from SP and IEL of young and old mice to investigate the cytotoxic potential (**Figure 3A**). As shown in **Figures 3B, C**, although IEL CD4^+^ T cells alone showed low cytotoxic activity, old IEL CD3^+^ T cells showed higher cytotoxic activity against tumor organoids than that of young IEL CD3^+^ T cells. Interestingly, the number of old IEL CD3^+^ T cells harvested after the co-incubation was much higher than that of young IEL CD3^+^ T cells (**Figure 3D**). We also confirmed the induction of high levels of MHC-II and PD-L1 on the EpCAM^+^ tumor epithelial cells co-cultured with IEL CD4 or CD3^+^ T cells (**Supplementary Figure 3**). These results suggest that IEL T cells exerted the strong immune response against tumor organoids, and that old IEL CD4^+^ T cells exert their anti-tumor activity through MHC-II dependent antigen presentation with the help of other T cell fractions such as γδT or CD8^+^ T cells.

### 3.5 Two distinct, unique CD4^+^ T cell subsets were increased in the small intestinal intraepithelial lymphocytes of aged mice

These results showed that although aging intestinal CD4^+^ T cells have similar characteristics to previously reported aging systemic T cells, such as reduced expression of co-stimulatory molecules and reduced proliferative capacity, they also have characteristics that are clearly distinct from those of aging systemic T cells, such as low SASP, high cytotoxicity and CD8αα expression. On the other hand, functional analysis of the bulk IEL CD4^+^ T cells showed limited results.

Therefore, we considered it difficult to understand the characteristics of old IEL CD4^+^ T cells by bulk CD4^+^ T cell analysis, and therefore, single cell analysis was performed with the aim of subdividing aging T cells and precisely studying their characteristics at the single cell level. CD3^+^ T cells were collected from the SP and IEL of young and old mice and sorted by flow cytometry, and clustering by scRNAseq, expressed gene analysis and T cell receptor repertoire analysis were performed. In young IEL CD4^+^ T cells, which are the focus of this study, are a minor fraction, and in order to analyze a sufficient number of cells, CD4^+^ T cells were enriched in both young and old IEL.

**Figure 4:**
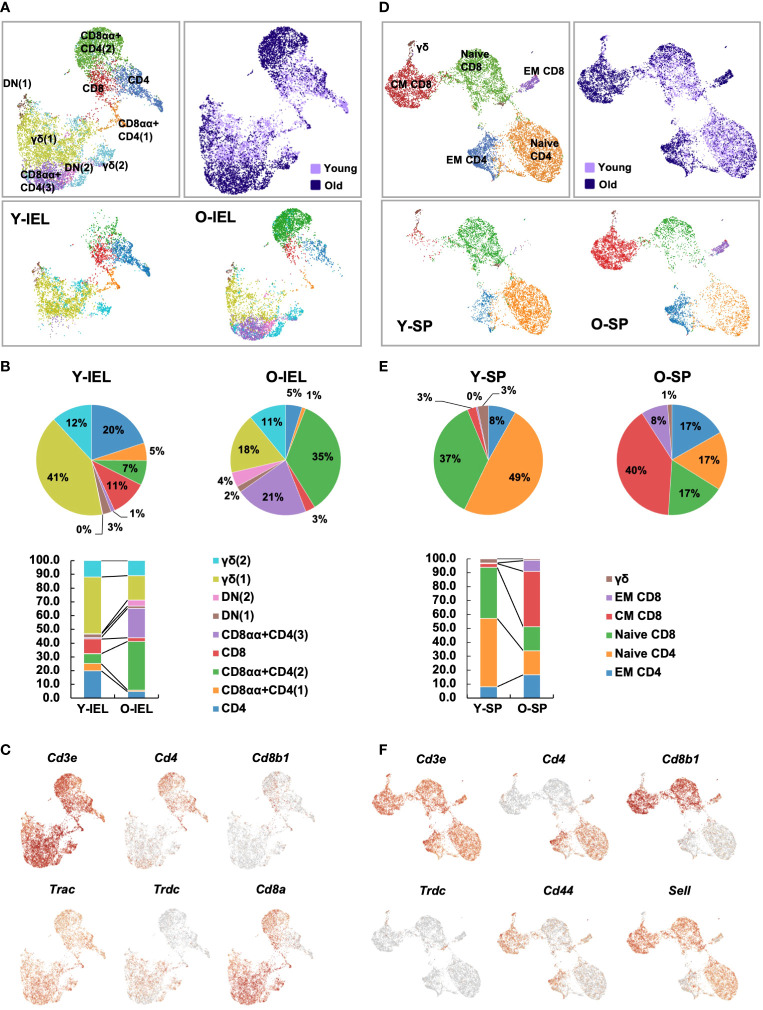
Single-cell gene expression analysis of SP and IEL T cells. SI-IELs or SP cells were collected and pooled from young (6-weeks old) or old (80-weeks old) C57BL/6 mice. The cells from 6 young mice and 3 old mice were mixed and pooled as young SP, young IEL, old SP and old IEL. To enrich CD4^+^ cells, which are a rare population in young IEL, CD3^+^ cells and CD3^+^CD4^+^ cells were mixed as 1:1 in young and old IEL. **(A)** UMAP projection of young and old IEL T cells. **(B)** The proportion of each cluster in IEL T cells. **(C)** Expression of canonical marker genes to define each cluster in IEL T cells. **(D)** UMAP projection of young and old SP T cells. The definition of each cluster is described in the first column. **(E)** The proportion of each cluster in SP T cells. **(F)** Expression of canonical marker genes to define each cluster in SP T cells.

In the UMAP of all T cells, IEL T cells were classified into 9 clusters (**Figure 4A**). Based on the expression pattern of each marker gene, it was confirmed that all IEL T cells analyzed were CD3^+^ T cells, and each cluster was subdivided into CD4, CD8, γδ and DNT; CD4 was further subdivided into CD8αα^+^CD4 and CD8αα^-^CD4 (**Figures 4A–C** and **Supplementary Figure 4**). SP T cells were grouped into 6 clusters (**Figures 4D–F**). Based on the expression of each marker gene, splenic T cells were classified as naïve CD4, naïve CD8, central memory CD8, effector memory CD8, effector memory CD4 and γδ T cells (**Figures 4D–F** and **Supplementary Figure 4**). As previously reported, young SP were predominantly naïve, whereas old SP showed a marked increase in memory CD4 and CD8 (**Figures 4D, E**). Interestingly, comparison of old and young IEL showed that they were quite different, with young IEL being dominated by CD4, CD8 and γδ T cells, whereas old IEL showed the appearance of age-specific CD8αα^+^CD4^+^ T cell clusters, whereas in SP, most of the fractions were overlapped between young and old T cells (**Figures 4A, B**).

**Figure 5:**
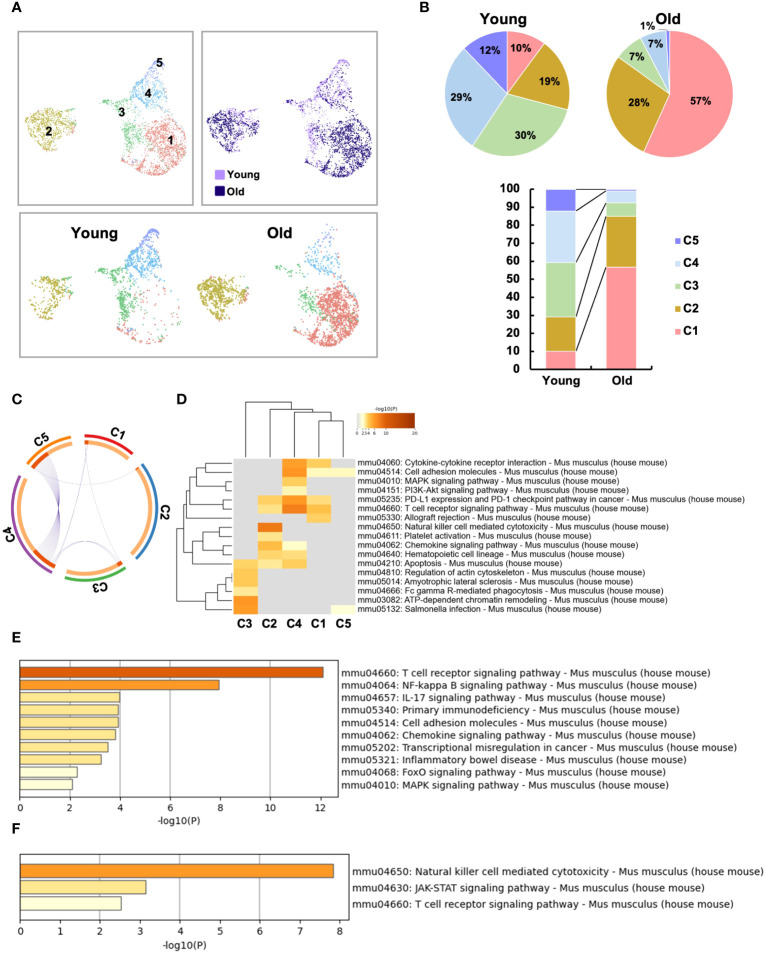
Two distinct, unique CD4^+^ T cell subsets were increased in the small intestinal intraepithelial lymphocytes of aged mice. To further analyze the age-related changes in the IEL CD4 fraction, re-clustering was performed. **(A)** UMAP projection of sub-clustered IEL CD4^+^ T cells from young and old mice. **(B)** The proportion of each cluster in IEL CD4^+^ T cells from young and old mice. **(C)** The Circos plot shows how genes from the input gene lists overlap. Inside, each arc represents a gene list, with each gene member of that list is assigned a point on the arc. Dark orange color represents the genes that are shared by multiple lists and light orange color represents genes that are unique to that gene list. Purple lines connect the same gene that is shared by multiple gene lists. **(D)** KEGG pathway analysis of genes enriched in each cluster. Heatmap cells are colored based on the P-values of the enriched terms, and white cells indicate the lack of enrichment for that term. **(E)** KEGG pathway analysis of genes enriched in cluster1 compared to cluster2. **(F)** KEGG pathway analysis of genes enriched in cluster2 compared to cluster1.

To further analyze the aging-related changes in the IEL CD4 fraction, re-clustering was performed and revealed that IEL CD4^+^ T cells fell into five clusters (**Figure 5A**). Old IEL CD4^+^ T cells were broadly subdivided into two clusters, C1 and C2; C2 was also present in young IEL CD4^+^ T cells, albeit at a lower proportion, whereas C1 was found to be an old IEL CD4^+^ T cell specific fraction (**Figures 5A, B**). According to the pathway analysis, C1 and C2 are independent of each other, whereas C4 and C5 or C3 and C4 had some association (**Figure 5C**). Pathway analysis also revealed that although the NK-kB signaling, Th17, and chemokine signaling pathways were enriched in C1 subset compared to C2, the NK cell-mediated cytotoxicity and JAK-STAT signaling pathways were enriched in C2 subset compared to C1 (**Figures 5D–F**).

**Figure 6:**
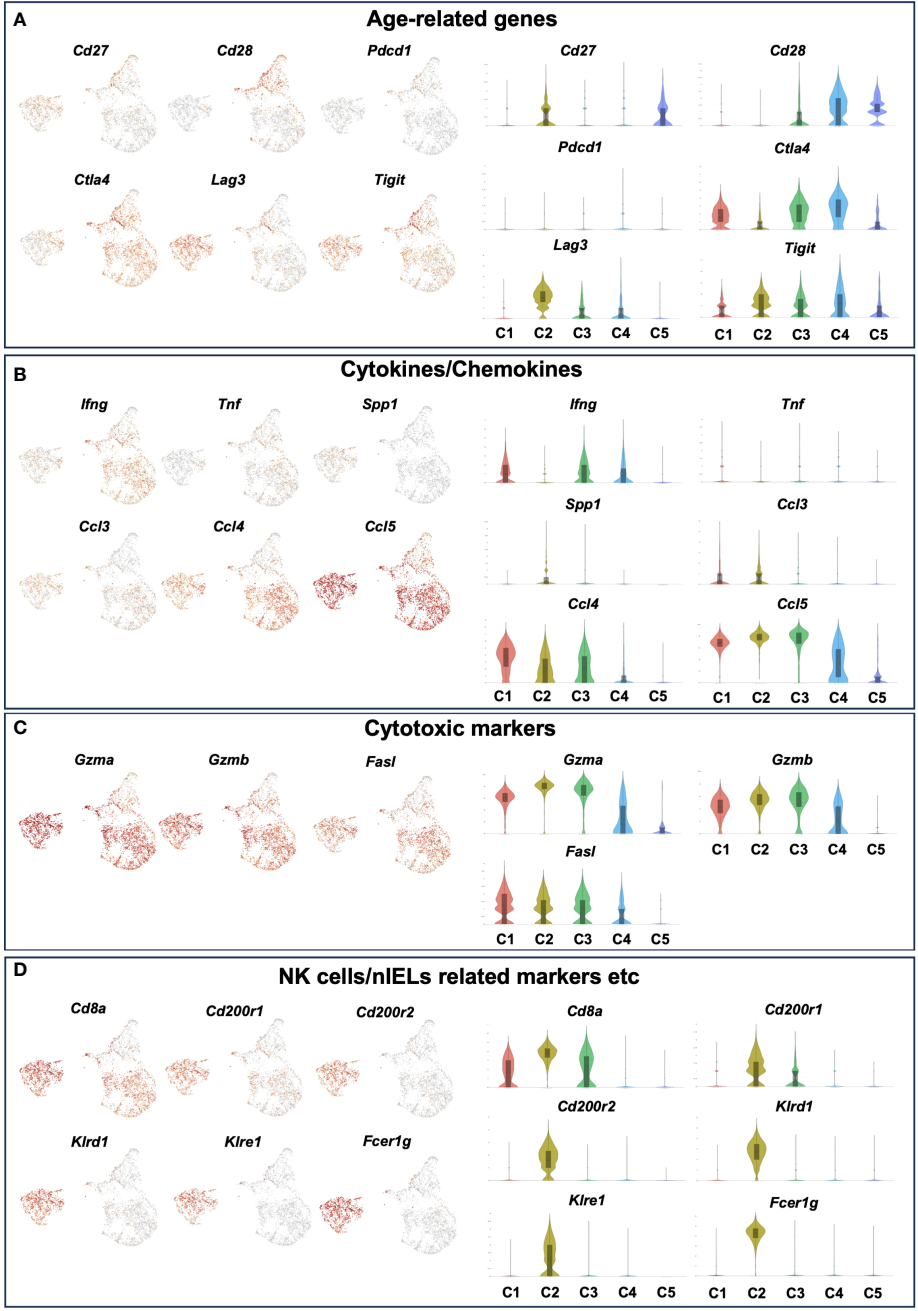
The expression level of age-related genes, cytokines/chemokines, cytotoxic markers and NK cells/nIELs related genes in each cluster of young and old IEL CD4^+^ T cells. Gene expression of each cluster of young and old IEL CD4^+^ T cells defied in **Figure 5**. **(A)** Age-related genes. **(B)** Cytokine/Chemokine genes. **(C)** Cytotoxic marker genes. **(D)** NK cells/nIELs related genes and others.

We therefore defined C1 as age-related IEL CD4^+^ T cell subset 1, and C2 as age-related IEL CD4^+^ T cell subset 2, and noticed their gene expression. In terms of age-related genes, although both didn’t express *Cd28*, subset 2 expressed low levels of *Cd27*. Subset 1 and other clusters expressed *Ctla4*, whereas subset 2 didn’t express it but expressed high levels of *Lag3* and *Tigit* (**Figure 6A** and **Supplementary Figure 5A**).

Regarding cytokines and chemokines, subset 1 expressed *Ifng*, whereas subset 2 didn’t express it. Both subsets didn’t express *Tnf*, which is consistent with the result of the bulk CD4^+^ T cell analysis (**Figure 2E**). Interestingly, both subsets expressed the chemokines *Ccl3, Ccl4* and *Ccl5* (**Figure 6B**).

In terms of cytotoxic markers, both subsets expressed high levels of *Gzma, Gzmb*, but subset 2 expressed higher levels of them than subset 1. Both expressed *Fasl* (**Figure 6C** and **Supplementary Figure 5B**).

Although both subsets expressed high levels of *Cd8a*, subset 2 expressed higher levels of it than subset 1 (**Figure 6D**). Interestingly, subset 2 expressed very unique genes such as *Cd200r1, Cd200r2, klrd1, klre1, Tnfrsf9* and *Fcer1g*, which are features of natural IEL (**Figure 6D** and **Supplementary Figure 5C**). Both subsets didn’t express Treg marker genes such as *foxp3* and *Il10*, or Th17 marker genes such as *Il17a* and *Il21* (**Supplementary Figure 5D**).

**Figure 7:**
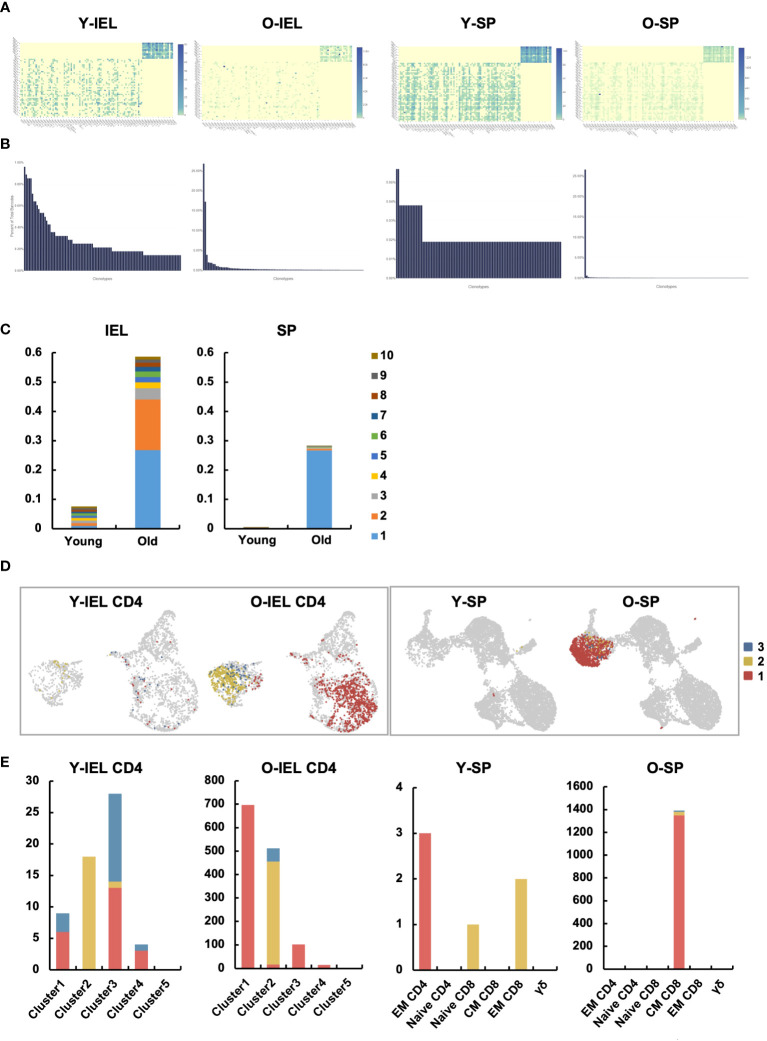
Two distinct age-related IEL CD4^+^ T cell subsets showed very specific TCR chronotypes that differed each other. TCR repertoire analysis was performed to determine age-related changes in T cell clonality in IEL and SP. **(A)** Heatmaps show the expression frequency of V-J gene pairs in young IEL (Y-IEL), old IEL (O-IEL), young SP (Y-SP) and old SP T cells (O-SP). The x and y axes indicate the combination of TRA + BV and TRA + BJ genes. **(B)** The graphs show the percentage of each TCR clonotype in each group. **(C)** The frequency of the top 10 TCR clonotypes in each group. **(D)** UMAP shows the distribution of the top 3 clonotypes in each group. **(E)** The graphs show the numbers of the top 3 clonotypes in each group.

TCR repertoire analysis was performed to determine age-related changes in T cell clonality in IEL and SP. Diversity was predominantly reduced at SP (**Figures 7A–C** and **Supplementary Figure 6**), as previously reported (28, 29). In the gut, diversity was also significantly reduced, with less than 22 of the top chronotypes showing diversity in young IEL, whereas in old IEL, two chronotypes formed the majority, and the frequency of the others was significantly reduced (**Figures 7A–C** and **Supplementary Figure 6**). Interestingly, the most abundant chronotype (830) was found to be expressed in most of subset 1, whereas the second most abundant chronotype (439) was found to be expressed only in subset 2. In contrast, the old SP chronotype was concentrated in one clonotype, and this TCR was concentrated in the central memory CD8 (**Figures 7D, E**).

## 4 Discussions

The intestinal tract, the frontline of enormous foreign antigens such as food antigens and gut microbiota, is always in a dynamic balance between inflammation and tolerance and has a highly developed and unique immune system to maintain homeostasis, particularly unique are the IELs; the number of IELs is comparable to the total number of T cells in the spleen and includes T cell fractions unique to the gut, γδ T cells and DNT cells. These fractions, also known as natural IELs (nIELs) because they are abundant immediately after birth, have a limited TCR repertoire and express many innate immune markers such as NK receptors and are thought to maintain epithelial cell homeostasis. On the other hand, major fractions in the peripheral blood, such as CD4^+^ T cells and CD8^+^ T cells, are also referred to as induced IELs (iIELs).

The intestinal tract is an organ that is relatively unaffected by aging compared to the skin, hair and vasculature. Although reduced barrier function (30), increased susceptibility to DSS enteritis (31), reduced stem cell function (32–35) and shortened villi (34) have been reported, there are few reports of changes in the immune system.

In the present study, we found a marked increase in intestinal T cells, particularly CD4^+^ T cells in the small intestinal IEL. Previous reports have suggested that nIELs decrease and iIELs increase with age. However, in aged mice, there was no significant change in the number of γδ and DNT cells, while CD4^+^ T cells increased but CD8^+^ T cells did not increase. These aging IEL CD4^+^ T cells were CD27^-^CD28^-^ like so-called aged systemic T cells, but differed in many ways. Their proliferative capacity was significantly lower than that of SP CD4^+^ T cells, they had a reduced capacity to produce inflammatory cytokines such as TNF-α, and they expressed surface markers similar to nIELs, such as NK receptors, granzymes and CD8αα.

Based on these characteristics, we hypothesized that old IEL CD4, like nIELs and CD8αα^+^CD4^+^ T cells, may play an important role in anti-tumor immunity and intestinal immune homeostasis via cytotoxic activity, and performed functional analysis *in vitro.* Although IEL CD4^+^ T cells alone showed low cytotoxic activity, old IEL CD3^+^ T cells showed higher cytotoxic activity against tumor organoids than that of young IEL CD3^+^ T cells. The results of the scRNAseq analysis showed that the aged intestinal CD4^+^ T cells, which had been analyzed together were actually an aggregate of two fractions with completely different properties. Subset 1 has a strong pro-inflammatory component, with secretion of *Ifng* and *Ccl4*, and TCR and NFkB signals being extracted in pathway analysis, while expression of inhibitory molecules such as *Ctla4* is also observed. Subset 2 has no *Ifng* expression, high expression of inhibitory molecules such as *Lag3* and *Tigit*, nIEL markers such as *NKR, Cd200r* and *Fcer1g*, and a strong tolerogenic element, while *Ccl5* expression is high, and *granzyme, Cd8a*, which is characteristic of aging intestinal CD4^+^ T cells expression was common to both fractions. These two fractions were in opposite positions in the clustering by UMAP and indeed had different repertoires. This may explain why the cytotoxic capacity of the bulk CD4^+^ T cells alone was weak. Although it is difficult to infer the function of each fraction simply from the genes expressed, the results suggest that they at least have properties that are very different from the aging T cells of the systemic immune system.

CD4^+^ T cells with cytotoxic properties have recently attracted attention (36–38). These cells are increased in the peripheral blood of super-centenarians, suggesting that they play an important role in the immune system of long-lived individuals (39, 40). In addition, innate T cell fractions expressing high levels of *Fcer1g* have recently been reported to play an important role in tumor immunity (41). As subset 2, the aging- specific subset, expresses high levels of *granzymes* and *Fcer1g*, they may have cytotoxic potential in tumor immunity with an increased risk of developing tumors with age. In addition, as they express nIEL-like surface markers such as NK receptors, they may play an important role not only in anti-tumor immunity but also in epithelial cell quality control, such as eliminating stress cells and maintaining barrier function in aged individuals.

Old IEL CD4^+^ T cells expressed high levels of the gut-specific marker CD8αα homodimer. CD8αα^+^CD4^+^ T cells have been reported to play an immunosuppressive role in IBD (42–45). It is known that there are fewer Foxp3^+^ regulatory T cells (Treg) in the small intestine than in the large intestine, and pathways for Treg differentiation into CD4^+^ IELs have also been reported (46). Old IEL CD4^+^ T cells, particularly subset 2, which express high levels of tolerogenic markers and CD8αα may induce immune tolerance, in contrast to systemic aged T cells that secrete pro-inflammatory cytokines and induce chronic inflammation.

Although the present study did not examine humans, cytotoxic CD4^+^ T cells are known to be increased in the peripheral blood of human long-lived human subjects (39, 40), suggesting an association with intestinal CD4^+^ T cells. Comparisons of the proportion of subset 1/2 in different elderly people are also of interest.

In the present study, although we attempted to deplete IEL CD4^+^ T cells with an anti-CD4 depletion antibody to verify the physiological role of aging IEL CD4^+^ T cells, they could not be deleted by commonly used clones. Adoptive transfer into RAG deficient mice was also attempted. Although young IEL CD4^+^ T cells were successfully engrafted, it was difficult to engraft old IEL CD4^+^ T cells with their low proliferative capacity.

Although we have also tried to sort subset 1 and subset 2 from SI-IEL of aged mice, we could not separate them due to a large discrepancy between the scRNAseq results and the flow cytometry results, with some mRNAs expressed but no surface markers stained, and others with low mRNA expression but surface markers stained (data not shown). In the future, we would like to investigate the physiological role of these cells by generating mice specifically deficient in subset 1 and subset 2 by creating appropriate conditioned KO mice or appropriate reporter mice.

In the current study, we discovered for the first time that two unique CD4^+^ T cell subsets, distinct from the aging systemic T cells, are increased in the intestinal mucosa of aged mice. Subset 1 has a strong pro-inflammatory component, with expression of *Ifng* and *Ccl4* and upregulation of TCR and NFkB signaling pathways. Subset 2, on the other hand, does not express *Ifng*, but upregulates inhibitory molecules such as *Lag3* and *Tigit*, and nIEL markers such as *NKR*, *Cd200r* and *Fcer1g*. Expression of *granzymes* and *Cd8a* was common to both. These two fractions were in opposite positions in the clustering by UMAP and indeed had different TCR repertoires.

Although the detailed functions of these fractions are still unknown, they may be involved in the suppression of intestinal aging and longevity through anti-tumor immunity, elimination of senescent cells and stressed cells in the aging environment. This finding could be a breakthrough in aging research.

## Data availability statement

The data presented in this study are deposited in the GEO repository under the following accession numbers (microarray: “GSE249501”; scRNAseq: “GSE252360”).

## Ethics statement

The animal study was approved by Center for Experimental Animals in Tokyo Medical and Dental University. The study was conducted in accordance with the local legislation and institutional requirements.

## Author contributions

YY: Conceptualization, Investigation, Writing – review & editing. YN: Conceptualization, Investigation, Funding acquisition, Writing – original draft. RM: Investigation, Writing – review & editing. NS: Writing – review & editing. SO: Conceptualization, Funding acquisition, Writing – review & editing. TN: Conceptualization, Funding acquisition, Writing – review & editing. TM: Conceptualization, Writing – review & editing. GI: Conceptualization, Writing – review & editing. SF: Conceptualization, Writing – review & editing. RO: Conceptualization, Funding acquisition, Supervision, Writing – review & editing.

## References

[1] Collaborators GDaH. Global, regional, and national disability-adjusted life-years (DALYs) for 315 diseases and injuries and healthy life expectancy (HALE), 1990-2015: a systematic analysis for the Global Burden of Disease Study 2015. Lancet (2016) 388(10053):1603–58. doi: 10.1016/S0140-6736(16)31460-X PMC5388857 27733283

[2] Jurk D Wilson C Passos JF Oakley F Correia-Melo C Greaves L. Chronic inflammation induces telomere dysfunction and accelerates aging in mice. Nat Commun (2014) 2:4172. doi: 10.1038/ncomms5172 24960204 PMC4090717

[3] Di Micco R Fumagalli M Cicalese A Piccinin S Gasparini P Luise C. Oncogene-induced senescence is a DNA damage response triggered by DNA hyper-replication. Nature (2006) 444(7119):638–42. doi: 10.1038/nature05327 17136094

[4] Desdín-Micó G Soto-Heredero G Aranda JF Oller J Carrasco E Gabandé-Rodríguez E. T cells with dysfunctional mitochondria induce multimorbidity and premature senescence. Science (2020) 368(6497):1371–6. doi: 10.1126/science.aax0860 PMC7616968 32439659

[5] Ristow M Zarse K. How increased oxidative stress promotes longevity and metabolic health: The concept of mitochondrial hormesis (mitohormesis). Exp Gerontol (2010) 45(6):410–8. doi: 10.1016/j.exger.2010.03.014 20350594

[6] Schriner SE Linford NJ Martin GM Treuting P Ogburn CE Emond M. Extension of murine life span by overexpression of catalase targeted to mitochondria. Science (2005) 308(5730):1909–11. doi: 10.1126/science.1106653 15879174

[7] Dillin A Hsu AL Arantes-Oliveira N Lehrer-Graiwer J Hsin H Fraser AG. Rates of behavior and aging specified by mitochondrial function during development. Science (2002) 298(5602):2398–401. doi: 10.1126/science.1077780 12471266

[8] Houtkooper RH Mouchiroud L Ryu D Moullan N Katsyuba E Knott G. Mitonuclear protein imbalance as a conserved longevity mechanism. Nature (2013) 497(7450):451–7. doi: 10.1038/nature12188 PMC3663447 23698443

[9] Bartkova J Rezaei N Liontos M Karakaidos P Kletsas D Issaeva N. Oncogene-induced senescence is part of the tumorigenesis barrier imposed by DNA damage checkpoints. Nature (2006) 444(7119):633–7. doi: 10.1038/nature05268 17136093

[10] Campisi J Kapahi P Lithgow GJ Melov S Newman JC Verdin E. From discoveries in aging research to therapeutics for healthy aging. Nature (2019) 571(7764):183–92. doi: 10.1038/s41586-019-1365-2 PMC7205183 31292558

[11] Ferrucci L Fabbri E. Inflammaging: chronic inflammation in aging, cardiovascular disease, and frailty. Nat Rev Cardiol (2018) 15(9):505–22. doi: 10.1038/s41569-018-0064-2 PMC6146930 30065258

[12] Rea IM Gibson DS McGilligan V McNerlan SE Alexander HD Ross OA. Age and age-related diseases: role of inflammation triggers and cytokines. Front Immunol (2018) 9:586. doi: 10.3389/fimmu.2018.00586 29686666 PMC5900450

[13] Palmer DB. The effect of age on thymic function. Front Immunol (2013) 4:316. doi: 10.3389/fimmu.2013.00316 24109481 PMC3791471

[14] Mittelbrunn M Kroemer G. Hallmarks of T cell aging. Nat Immunol (2021) 22(6):687–98. doi: 10.1038/s41590-021-00927-z 33986548

[15] Zhang J He T Xue L Guo H. Senescent T cells: a potential biomarker and target for cancer therapy. EBioMedicine (2021) 68:103409. doi: 10.1016/j.ebiom.2021.103409 34049248 PMC8170103

[16] Wang TW Johmura Y Suzuki N Omori S Migita T Yamaguchi K. Blocking PD-L1-PD-1 improves senescence surveillance and aging phenotypes. Nature (2022) 611(7935):358–64. doi: 10.1038/s41586-022-05388-4 36323784

[17] Fukushima Y Minato N Hattori M. The impact of senescence-associated T cells on immunosenescence and age-related disorders. Inflammation Regener (2018) 38:24. doi: 10.1186/s41232-018-0082-9 PMC6304761 30603051

[18] Yoshida S Nakagami H Hayashi H Ikeda Y Sun J Tenma A. The CD153 vaccine is a senotherapeutic option for preventing the accumulation of senescent T cells in mice. Nat Commun (2020) 11(1):2482. doi: 10.1038/s41467-020-16347-w 32424156 PMC7235045

[19] Cheroutre H. IELs: enforcing law and order in the court of the intestinal epithelium. Immunol Rev (2005) 206:114–31. doi: 10.1111/j.0105-2896.2005.00284.x 16048545

[20] Cheroutre H Lambolez F Mucida D. The light and dark sides of intestinal intraepithelial lymphocytes. Nat Rev Immunol (2011) 11(7):445–56. doi: 10.1038/nri3007 PMC3140792 21681197

[21] Merideth MA Gordon LB Clauss S Sachdev V Smith AC Perry MB. Phenotype and course of Hutchinson-Gilford progeria syndrome. N Engl J Med (2008) 358(6):592–604. doi: 10.1056/NEJMoa0706898 18256394 PMC2940940

[22] Fujihashi K Kiyono H. Mucosal immunosenescence: new developments and vaccines to control infectious diseases. Trends Immunol (2009) 30(7):334–43. doi: 10.1016/j.it.2009.04.004 19540811

[23] Shimatani K Nakashima Y Hattori M Hamazaki Y Minato N. PD-1+ memory phenotype CD4+ T cells expressing C/EBPalpha underlie T cell immunodepression in senescence and leukemia. Proc Natl Acad Sci U.S.A. (2009) 106(37):15807–12. doi: 10.1073/pnas.0908805106 PMC2739871 19805226

[24] Krishnarajah S Ingelfinger F Friebel E Cansever D Amorim A Andreadou M. Single-cell profiling of immune system alterations in lymphoid, barrier and solid tissues in aged mice. Nat Aging (2022) 2(1):74–89. doi: 10.1038/s43587-021-00148-x 37118354

[25] Pálovics R Keller A Schaum N Tan W Fehlmann T Borja M. Molecular hallmarks of heterochronic parabiosis at single-cell resolution. Nature (2022) 603(7900):309–14. doi: 10.1038/s41586-022-04461-2 PMC9387403 35236985

[26] Villeda SA Plambeck KE Middeldorp J Castellano JM Mosher KI Luo J. Young blood reverses age-related impairments in cognitive function and synaptic plasticity in mice. Nat Med (2014) 20(6):659–63. doi: 10.1038/nm.3569 PMC4224436 24793238

[27] Morikawa R Nemoto Y Yonemoto Y Tanaka S Takei Y Oshima S. Intraepithelial lymphocytes suppress intestinal tumor growth by cell-to-cell contact via CD103/E-cadherin signal. Cell Mol Gastroenterol Hepatol (2021) 11(5):1483–503. doi: 10.1016/j.jcmgh.2021.01.014 PMC8025200 33515805

[28] Egorov ES Kasatskaya SA Zubov VN Izraelson M Nakonechnaya TO Staroverov DB. The changing landscape of naive T cell receptor repertoire with human aging. Front Immunol (2018) 9:1618. doi: 10.3389/fimmu.2018.01618 30087674 PMC6066563

[29] Shifrut E Baruch K Gal H Ndifon W Deczkowska A Schwartz M. CD4(+) T cell-receptor repertoire diversity is compromised in the spleen but not in the bone marrow of aged mice due to private and sporadic clonal expansions. Front Immunol (2013) 4:379. doi: 10.3389/fimmu.2013.00379 24312094 PMC3832891

[30] Sovran B Hugenholtz F Elderman M Van Beek AA Graversen K Huijskes M. Age-associated impairment of the mucus barrier function is associated with profound changes in microbiota and immunity. Sci Rep (2019) 9(1):1437. doi: 10.1038/s41598-018-35228-3 30723224 PMC6363726

[31] Liu A Lv H Wang H Yang H Li Y Qian J. Aging increases the severity of colitis and the related changes to the gut barrier and gut microbiota in humans and mice. J Gerontol A Biol Sci Med Sci (2020) 75(7):1284–92. doi: 10.1093/gerona/glz263 32048723

[32] Nalapareddy K Zheng Y Geiger H. Aging of intestinal stem cells. Stem Cell Rep (2022) 17(4):734–40. doi: 10.1016/j.stemcr.2022.02.003 PMC9023768 35276089

[33] Nalapareddy K Nattamai KJ Kumar RS Karns R Wikenheiser-Brokamp KA Sampson LL. Canonical wnt signaling ameliorates aging of intestinal stem cells. Cell Rep (2017) 18(11):2608–21. doi: 10.1016/j.celrep.2017.02.056 PMC5987258 28297666

[34] He D Wu H Xiang J Ruan X Peng P Ruan Y. Gut stem cell aging is driven by mTORC1 via a p38 MAPK-p53 pathway. Nat Commun (2020) 11(1):37. doi: 10.1038/s41467-019-13911-x 31896747 PMC6940394

[35] Pentinmikko N Iqbal S Mana M Andersson S Cognetta AB Suciu RM. Notum produced by Paneth cells attenuates regeneration of aged intestinal epithelium. Nature (2019) 571(7765):398–402. doi: 10.1038/s41586-019-1383-0 31292548 PMC8151802

[36] Zhou C Qiu Y Yang H. CD4CD8αα IELs: they have something to say. Front Immunol (2019) 10:2269. doi: 10.3389/fimmu.2019.02269 31649659 PMC6794356

[37] Tanemoto S Sujino T Miyamoto K Moody J Yoshimatsu Y Ando Y. Single-cell transcriptomics of human gut T cells identifies cytotoxic CD4. Front Immunol (2022) 13:977117. doi: 10.3389/fimmu.2022.977117 36353619 PMC9639511

[38] Hoeks C Duran G Hellings N Broux B. When helpers go above and beyond: development and characterization of cytotoxic CD4. Front Immunol (2022) 13:951900. doi: 10.3389/fimmu.2022.951900 35903098 PMC9320319

[39] Covre LP Martins RF Devine OP Chambers ES Vukmanovic-Stejic M Silva JA. Circulating senescent T cells are linked to systemic inflammation and lesion size during human cutaneous leishmaniasis. Front Immunol (2018) 9:3001. doi: 10.3389/fimmu.2018.03001 30662437 PMC6328442

[40] Hashimoto K Kouno T Ikawa T Hayatsu N Miyajima Y Yabukami H. Single-cell transcriptomics reveals expansion of cytotoxic CD4 T cells in supercentenarians. Proc Natl Acad Sci U.S.A. (2019) 116(48):24242–51. doi: 10.1073/pnas.1907883116 PMC6883788 31719197

[41] Chou C Zhang X Krishna C Nixon BG Dadi S Capistrano KJ. Programme of self-reactive innate-like T cell-mediated cancer immunity. Nature (2022) 605(7908):139–45. doi: 10.1038/s41586-022-04632-1 PMC9250102 35444279

[42] Das G Augustine MM Das J Bottomly K Ray P Ray A. An important regulatory role for CD4+CD8 alpha alpha T cells in the intestinal epithelial layer in the prevention of inflammatory bowel disease. Proc Natl Acad Sci U.S.A. (2003) 100(9):5324–9. doi: 10.1073/pnas.0831037100 PMC154344 12695566

[43] Sarrabayrouse G Bossard C Chauvin JM Jarry A Meurette G Quévrain E. CD4CD8αα lymphocytes, a novel human regulatory T cell subset induced by colonic bacteria and deficient in patients with inflammatory bowel disease. PloS Biol (2014) 12(4):e1001833. doi: 10.1371/journal.pbio.1001833 24714093 PMC3979654

[44] Bousbaine D Fisch LI London M Bhagchandani P Rezende de Castro TB Mimee M. A conserved Bacteroidetes antigen induces anti-inflammatory intestinal T lymphocytes. Science (2022) 377(6606):660–6. doi: 10.1126/science.abg5645 PMC9766740 35926021

[45] Sarrabayrouse G Alameddine J Altare F Jotereau F. Microbiota-specific CD4CD8αα Tregs: role in intestinal immune homeostasis and implications for IBD. Front Immunol (2015) 6:522. doi: 10.3389/fimmu.2015.00522 26500657 PMC4597122

[46] Sujino T London M Hoytema van Konijnenburg DP Rendon T Buch T Silva HM. Tissue adaptation of regulatory and intraepithelial CD4^+^ T cells controls gut inflammation. Science (2016) 352(6293):1581–6. doi: 10.1126/science.aaf3892 PMC4968079 27256884

