# Supplementary figures and images for: Single cell analysis revealed that two distinct, unique CD4+ T cell subsets were increased in the small intestinal intraepithelial lymphocytes of aged mice
Source: Front Immunol. 2024 Jan 22;15:1340048. doi: 10.3389/fimmu.2024.1340048 (PMC10848332; doi:10.3389/fimmu.2024.1340048)

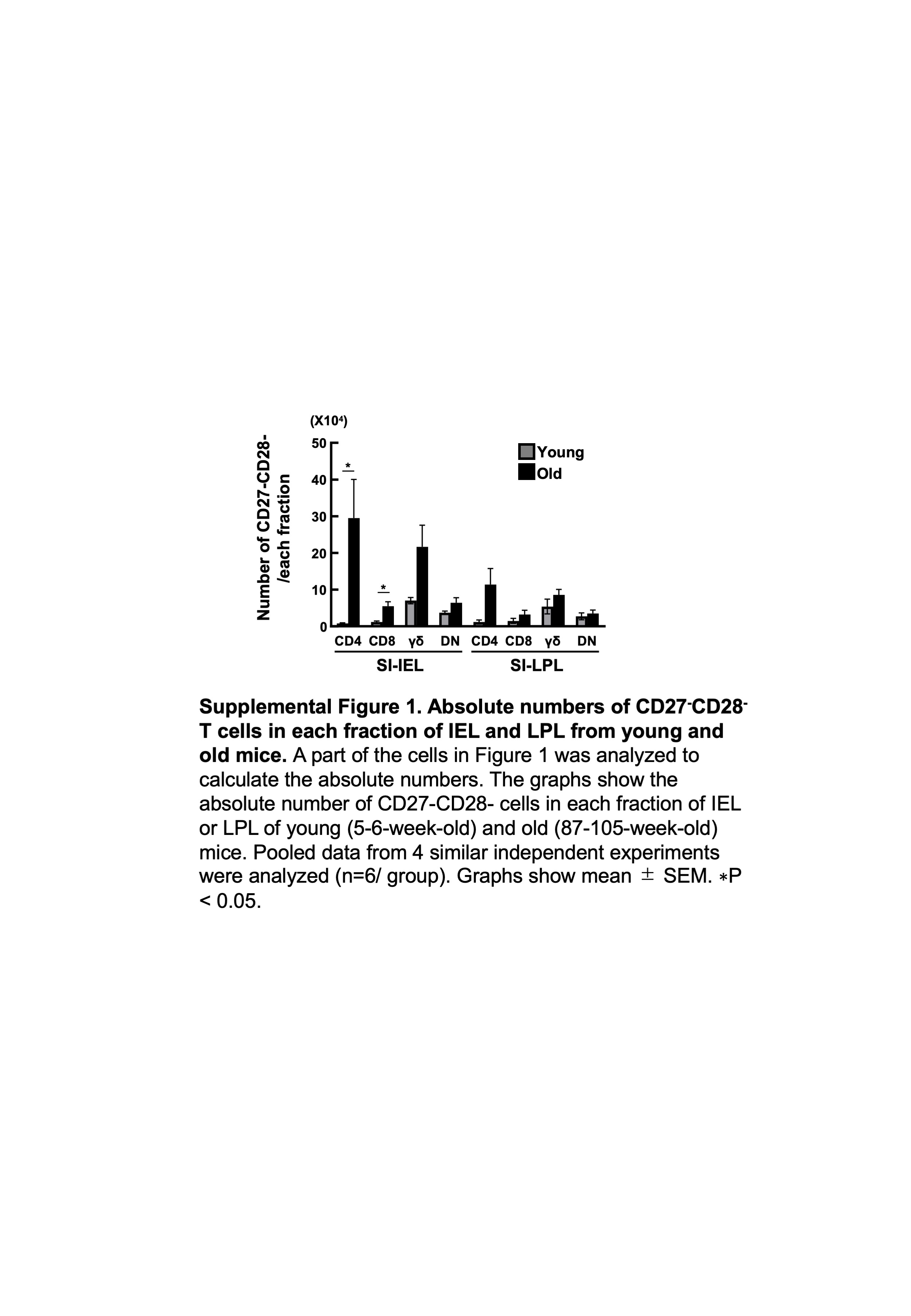

Supplement: Supplementary file 1 [file Image_1.jpeg]

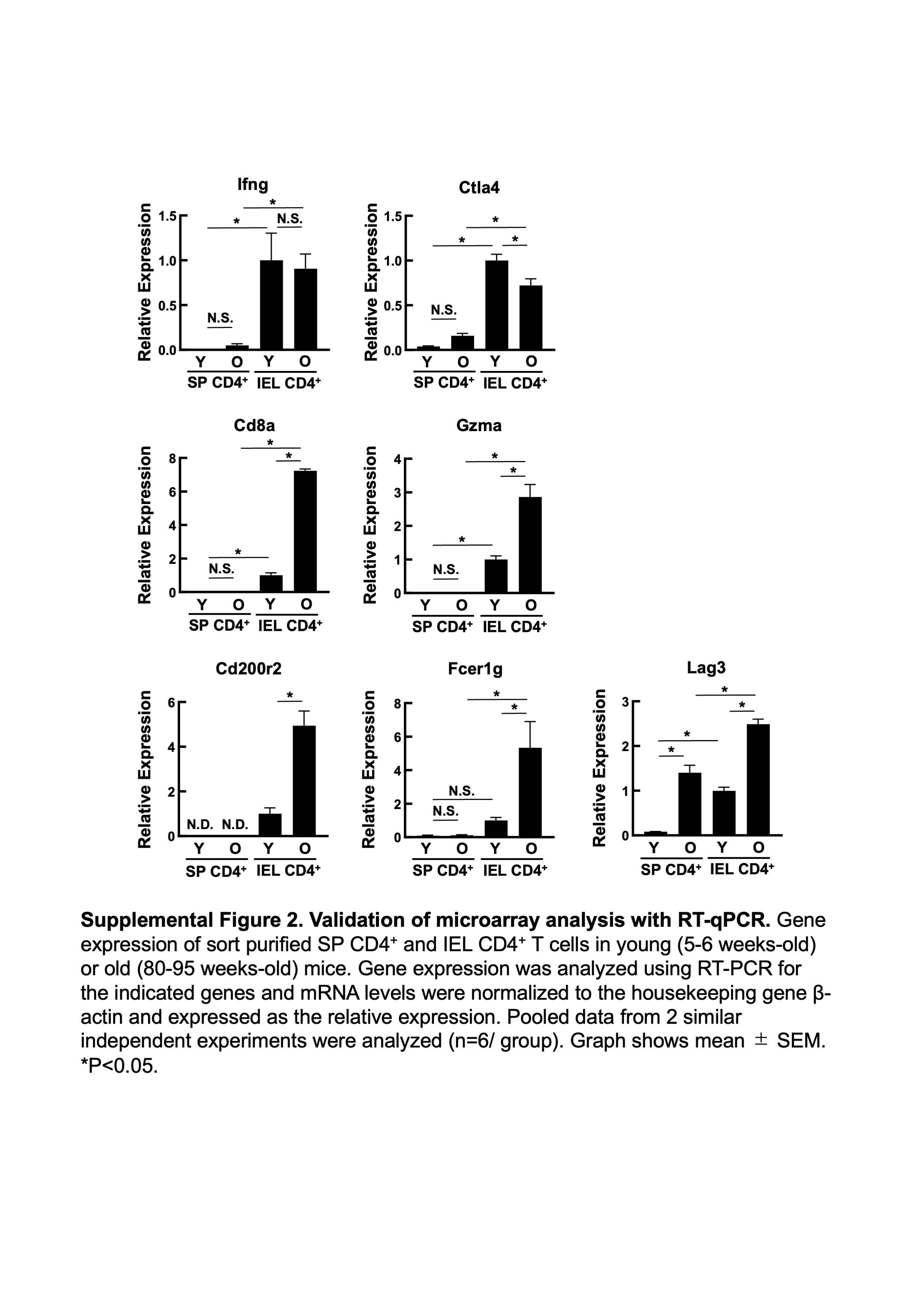

Supplement: Supplementary file 2 [file Image_2.jpeg]

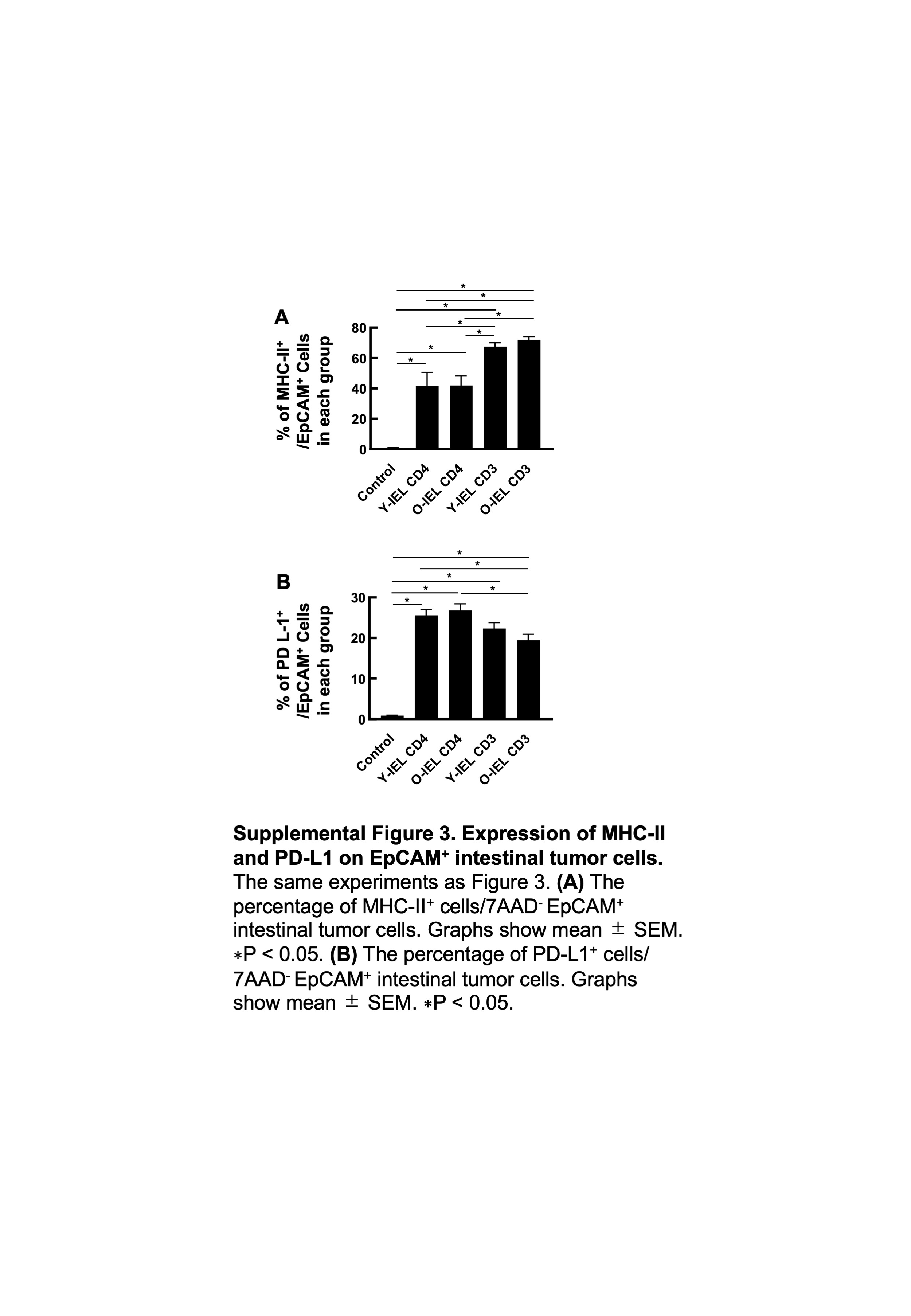

Supplement: Supplementary file 3 [file Image_3.jpeg]

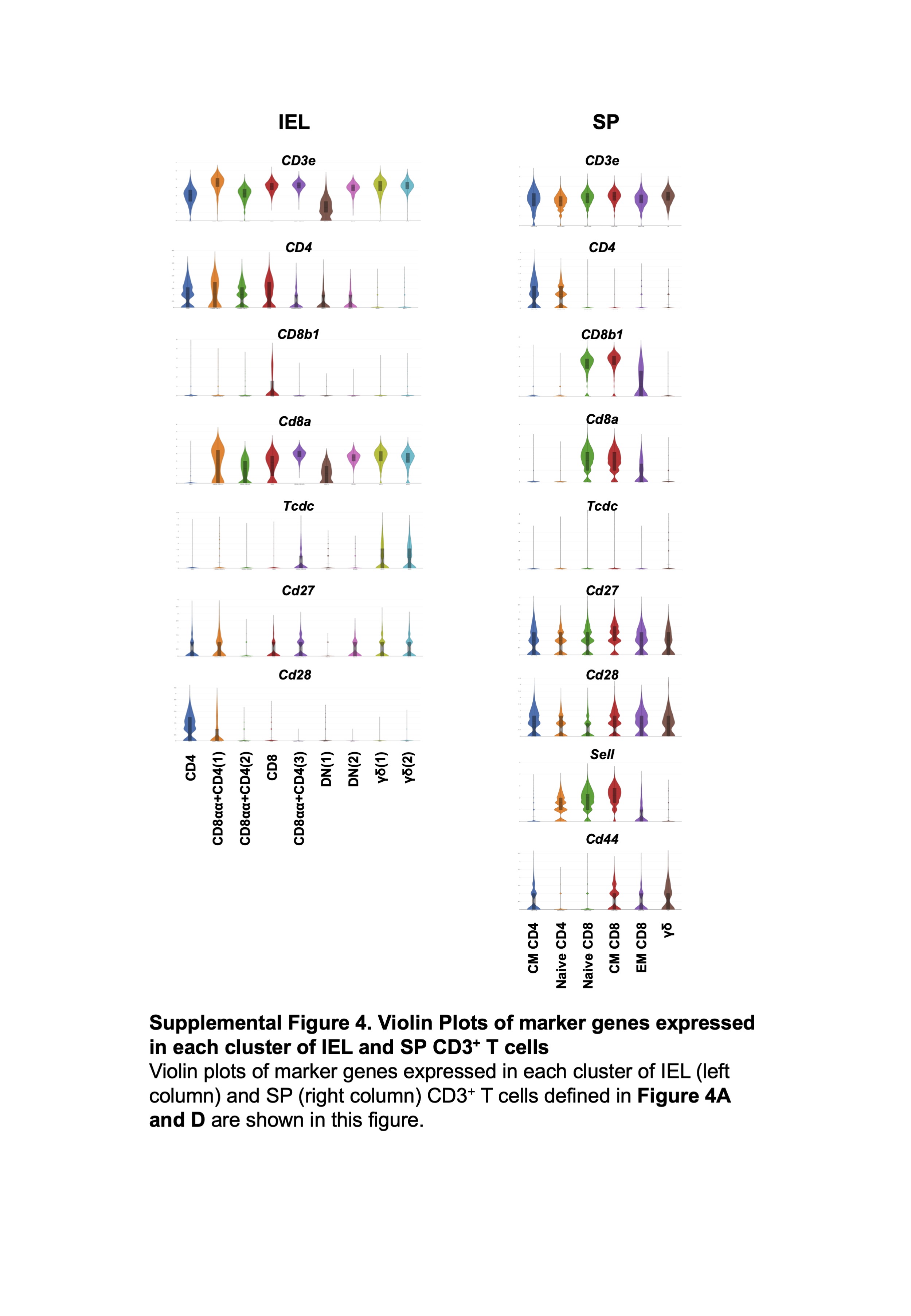

Supplement: Supplementary file 4 [file Image_4.jpeg]

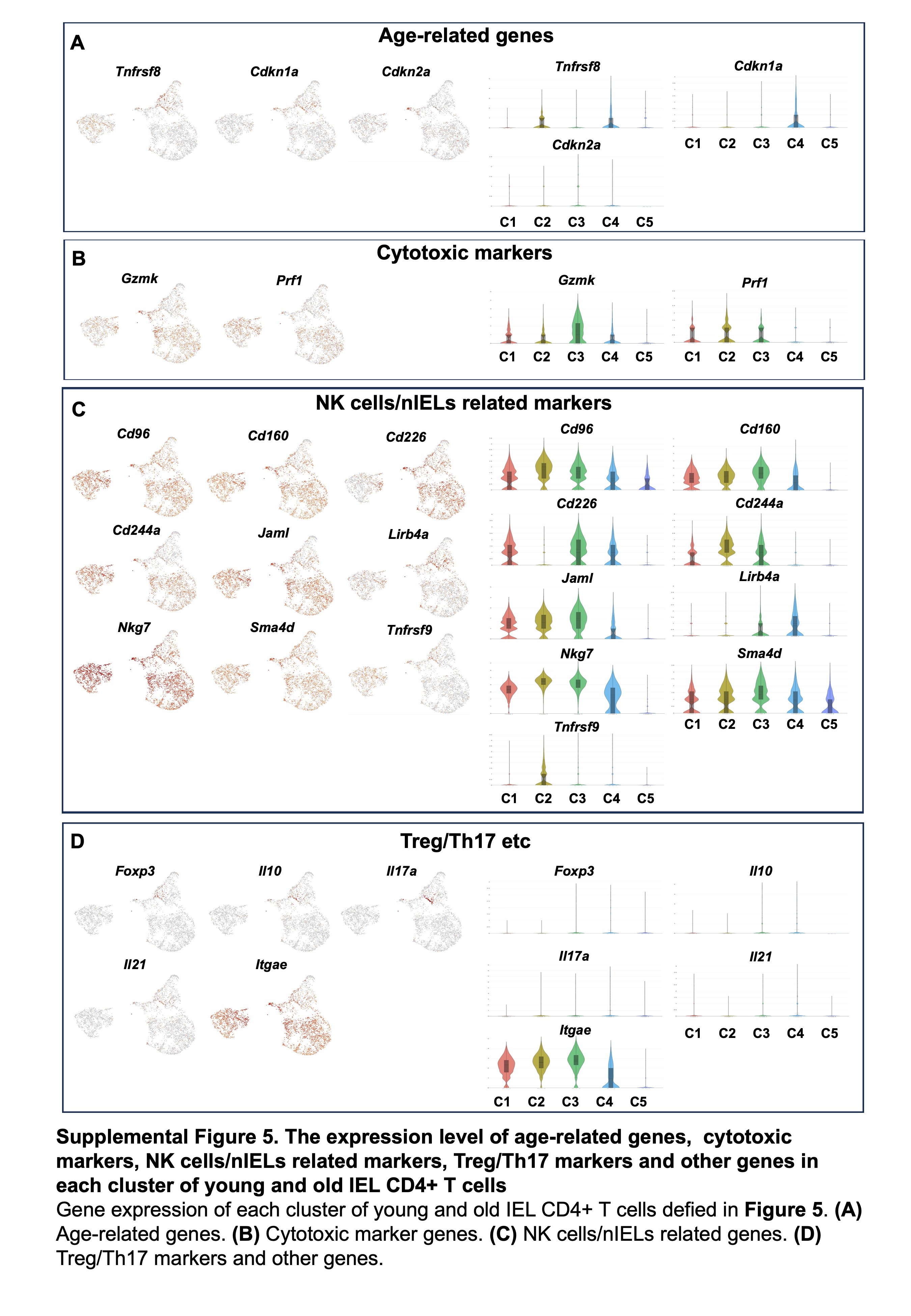

Supplement: Supplementary file 5 [file Image_5.jpeg]

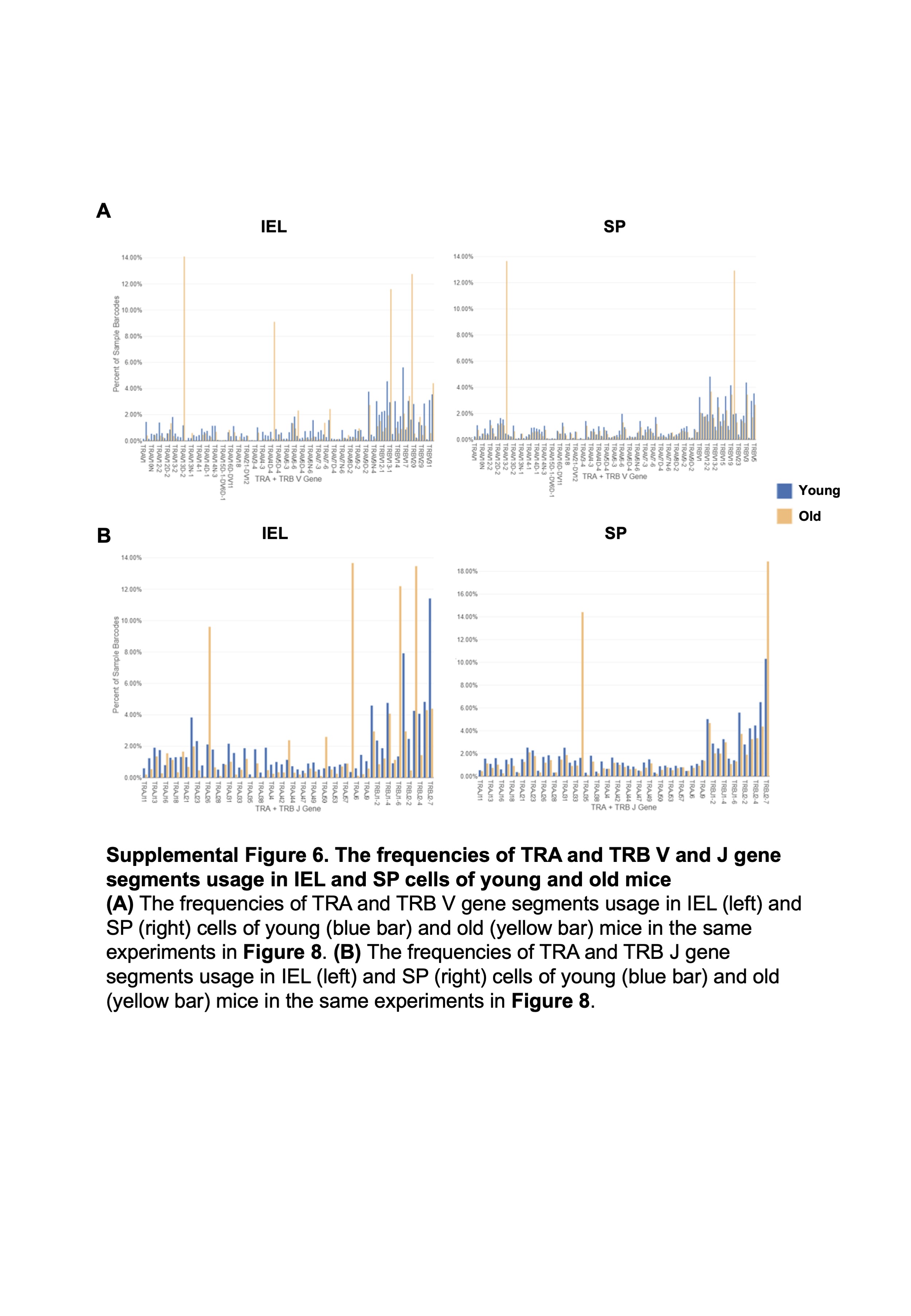

Supplement: Supplementary file 6 [file Image_6.jpeg]

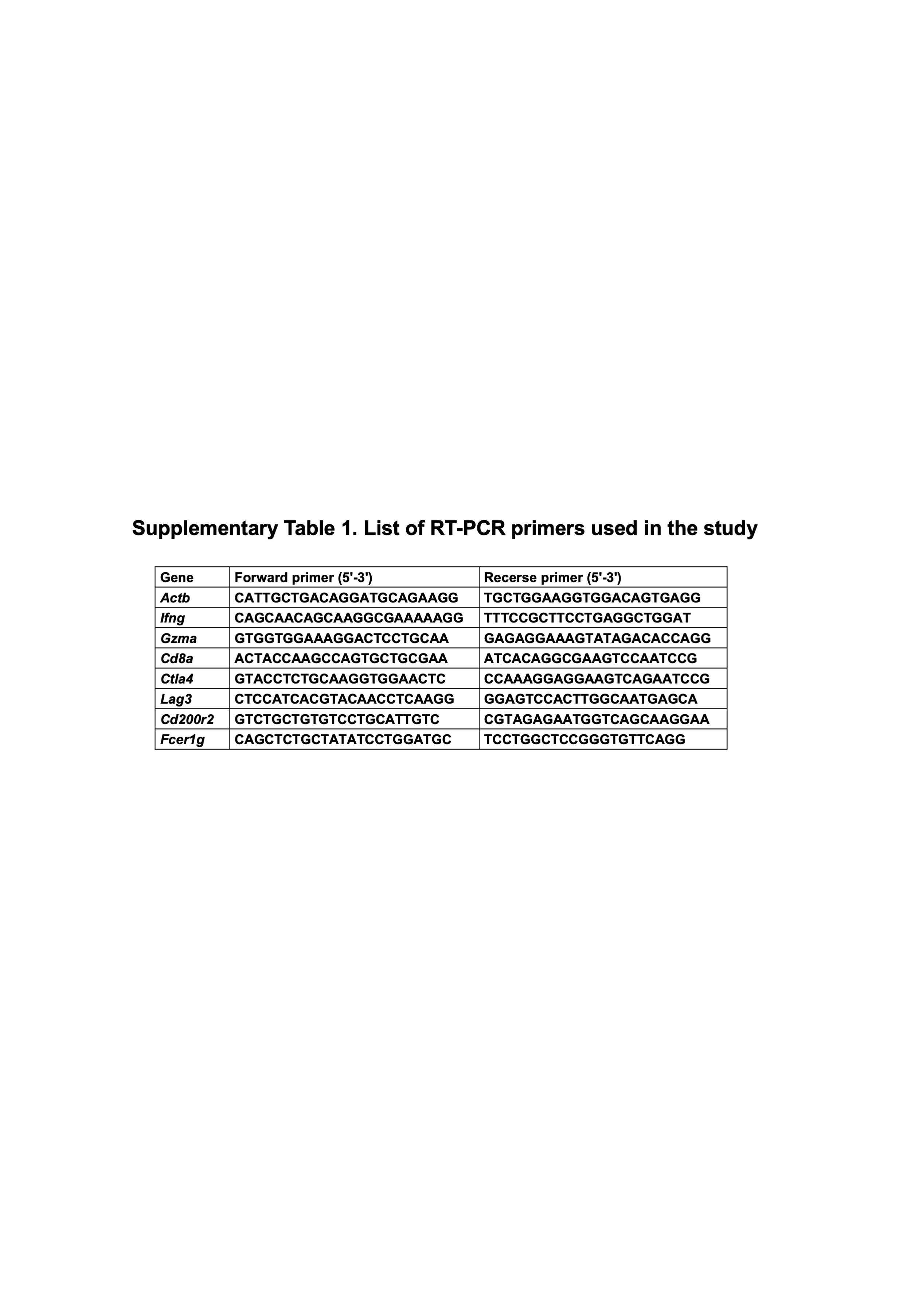

Supplement: Supplementary file 7 [file Image_7.jpeg]
